# Natural Isotopic Signatures of Variations in Body Nitrogen Fluxes: A Compartmental Model Analysis

**DOI:** 10.1371/journal.pcbi.1003865

**Published:** 2014-10-02

**Authors:** Nathalie Poupin, François Mariotti, Jean-François Huneau, Dominique Hermier, Hélène Fouillet

**Affiliations:** 1 INRA, CRNH-IdF, UMR914 Nutrition Physiology and Ingestive Behavior, Paris, France; 2 AgroParisTech, CRNH-IdF, UMR914 Nutrition Physiology and Ingestive Behavior, Paris, France; Johns Hopkins University, United States of America

## Abstract

Body tissues are generally ^15^N-enriched over the diet, with a discrimination factor (Δ^15^N) that varies among tissues and individuals as a function of their nutritional and physiopathological condition. However, both ^15^N bioaccumulation and intra- and inter-individual Δ^15^N variations are still poorly understood, so that theoretical models are required to understand their underlying mechanisms. Using experimental Δ^15^N measurements in rats, we developed a multi-compartmental model that provides the first detailed representation of the complex functioning of the body's Δ^15^N system, by explicitly linking the sizes and Δ^15^N values of 21 nitrogen pools to the rates and isotope effects of 49 nitrogen metabolic fluxes. We have shown that (i) besides urea production, several metabolic pathways (e.g., protein synthesis, amino acid intracellular metabolism, urea recycling and intestinal absorption or secretion) are most probably associated with isotope fractionation and together contribute to ^15^N accumulation in tissues, (ii) the Δ^15^N of a tissue at steady-state is not affected by variations of its P turnover rate, but can vary according to the relative orientation of tissue free amino acids towards oxidation vs. protein synthesis, (iii) at the whole-body level, Δ^15^N variations result from variations in the body partitioning of nitrogen fluxes (e.g., urea production, urea recycling and amino acid exchanges), with or without changes in nitrogen balance, (iv) any deviation from the optimal amino acid intake, in terms of both quality and quantity, causes a global rise in tissue Δ^15^N, and (v) Δ^15^N variations differ between tissues depending on the metabolic changes involved, which can therefore be identified using simultaneous multi-tissue Δ^15^N measurements. This work provides proof of concept that Δ^15^N measurements constitute a new promising tool to investigate how metabolic fluxes are nutritionally or physiopathologically reorganized or altered. The existence of such natural and interpretable isotopic biomarkers promises interesting applications in nutrition and health.

## Author Summary

Body proteins ensure vital functions, and their constancy is maintained through the tight coordination of many nitrogen metabolic fluxes, but our understanding of how this flux system is regulated, and sometimes dysregulated, remains fragmentary and incomplete. Besides, body tissues are generally naturally enriched in the heavier stable nitrogen isotope (^15^N) over the diet: this ^15^N bioaccumulation (Δ^15^N) varies depending on tissues and metabolic orientations, likely as the result of isotope effects associated to some metabolic pathways. We used a novel approach, combining multi-tissue Δ^15^N measurements and their analysis using modeling, to understand how body Δ^15^N values relate to nitrogen fluxes. The multi-tissue model we have developed provides a clearer understanding of the metabolic processes that generate isotopic fractionation, and of how tissue Δ^15^N values are modulated in response to changes in the body distribution of specific nitrogen fluxes. We show that Δ^15^N values tend to rise when the amino acids intake does not optimally fit the metabolic demand, and that Δ^15^N values constitute natural and interpretable signatures of nutritionally-induced variations in nitrogen fluxes. This approach constitutes a new promising tool to investigate how nitrogen metabolism is nutritionally or physiopathologically reorganized or altered, and promises interesting applications in many areas (nutrition, pathology, ecology, paleontology, etc).

## Introduction

Nitrogen (N) metabolism in the body involves a complex network of various between- and within-tissues fluxes, consisting in the transport and/or transformation of various N compounds such as proteins (P), amino acids (AA), urea and ammonia. These various N transfers and metabolic processes are critical to the tissue assimilation of dietary N, the elimination of some dietary and endogenous N (mostly as urinary urea) and continuous exchanges of N compounds between different body compartments, thus ensuring a body N balance and P homeostasis. In particular, within each tissue, P synthesis and degradation fluxes ensure a continuous turnover of the P mass, which is essential to preserve lean mass and the numerous vital functions of body P. Moreover, free AA are constantly exchanged between tissues and used for P synthesis within tissues where they also enter the transamination and deamination pathways (leading to the production of urea that is mostly excreted in urine) as well as other secondary metabolic pathways. All these N fluxes are closely coordinated and their regulation implies changes in their amplitude, orientation or distribution, in both the short term, in order to deal with the discontinuous dietary intakes (with daily cycles of fed and fasted states), and, in the long term, to adapt to changing nutritional or physiological conditions. However, dysregulation of this complex system of N fluxes may occur, involving metabolic perturbations, reorientations or imbalances and possibly leading to altered P homeostasis. Globally, little is still known about the inter-tissue N fluxes. The data available on different N fluxes are fragmented and dispersed because the classical investigative methods, mostly based on the administration of stable isotope-labelled metabolic tracers, usually focus on determining a specific type of flux (e.g., the administration of labelled AA to determine P synthesis rates, of labelled urea to study urea production and recycling fluxes, or of labelled dietary P to determine absorption kinetics, etc.) [1]–[4]. Because of these methodological limitations, we still have a poor understanding of the complex network of N metabolic fluxes between and within tissues, how they are coordinated and regulated in standard conditions and how they may be modulated as a function of changes in nutritional conditions (e.g., P intake) or dysregulated during a drift towards a pathological state. It is therefore necessary to develop new approaches that will provide an integrated insight into the whole-body distribution, partitioning and possible reallocations of N fluxes.

In parallel, a large body of evidence, mostly from ecological and archaeological studies, suggests that the N stable isotope compositions of metabolic pools (δ^15^N, the natural relative abundance of the rare stable isotope of N) are not only dependent on the δ^15^N of the diet but are also closely related to N metabolism and its modulations [5]. It is indeed well known that animal and human tissues are generally ^15^N-enriched relative to their diet, and several studies have reported that the extent of this tissue-to-diet ^15^N discrimination (Δ^15^N) varies between tissues in the same individual and may also differ between individuals depending on their particular nutritional or physiological conditions [5]–[8]. Such a δ^15^N trophic shift, and intra- and inter-individual Δ^15^N variations, are likely to result from the existence of isotope fractionation associated with certain metabolic pathways (e.g., deamination or transamination) that are sensitive to the isotope mass and use the two N isotopes at distinct rates [9]. When located at a metabolic branch point, isotope effects should theoretically lead to isotopic discrimination between the substrates and the competing products, with metabolic products having distinct isotopic values depending on the fractionation extent of the metabolic pathway from which they originate and the relative amplitude of the fluxes into the competing pathways [10]–[13]. Variations in the Δ^15^N of metabolic pools under specific nutritional or physiological conditions may therefore reflect underlying modulations in N fluxes, and, in particular, changes in the relative proportion of catabolism and anabolism and in the N balance [11], [14]–[16]. This remains however a general concept and the principal factors responsible for Δ^15^N and its variations, as well as the integrated functioning of the body Δ^15^N system, are still poorly understood. This raises important questions as to which metabolic processes are actually fractionating, how Δ^15^N differences between tissues are established and what relationships exist between variations in metabolic fluxes and actual Δ^15^N values [11], [17]. Furthermore, because of the complexity and intricacy of the metabolic pathways from which they originate, variations in Δ^15^N values cannot be interpreted directly in terms of the underlying rearrangements of body N fluxes that they might indicate. Mechanistic modeling approaches are therefore necessary to clarify the fractionating processes which lead to between-tissue Δ^15^N differences and to determine which variations in metabolic fluxes can explain between-subject Δ^15^N differences.

This study proposes a new approach that combines measurements of the size and Δ^15^N values of numerous N metabolic pools in rats and their analysis by compartmental modeling. We first developed a multi-compartmental model which reproduces AA and P metabolism in the whole body and which is consistent with previous data in the literature regarding the principal N metabolic flux values (absorption, excretion, urea production, P turnover, etc.) and known structural and dynamic system characteristics (metabolic compartmentation, differences in P turnover between tissues, etc.). Second, we showed that this model is able to reproduce the Δ^15^N variations observed experimentally among tissues and N fractions and thus to identify the fractionating processes that are responsible for such variations. Finally, model simulations enabled a clearer understanding of which flux modulations can impact tissue Δ^15^N values and, inversely, which, and in what way, Δ^15^N variations can reveal differences or rearrangements in N fluxes.

## Results

We developed a kinetic and mechanistic compartmental model of N metabolism which describes the relationships between different body N fluxes and natural N isotopic abundances in various pools. The model was built and calibrated based on experimental measurements of N amounts and isotopic abundances (δ^15^N) obtained under standard conditions in various body, dietary and elimination pools. The model calibration step resulted in the localization and quantification of the isotope fractionation associated with metabolic pathways. The calibrated model was then used for simulations to investigate the relationships between nutritionally- or physiopathologically-induced changes in N fluxes and ensuing variations in the isotopic signatures of body and elimination pools.

### Experimental evidence for variations in δ^15^N values between different tissues and fractions

**Figure 1:**
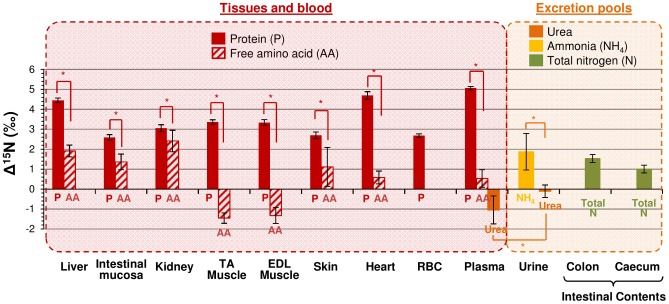
Natural nitrogen isotopic abundances of metabolic pools in rats under standard conditions. Isotopic abundances, measured in the protein (P) and free amino acid (AA) fractions of tissues and blood, in plasma urea, in urinary urea and ammonia (NH_4_), and in the total nitrogen (N) of intestinal contents, are expressed as the difference between the measured natural ^15^N enrichment of the pool and that of the diet (Δ^15^N_pool_ = δ^15^N_tissue_−δ^15^N_diet_). RBC, Red blood cells; TA, Tibialis anterior; EDL, Extensor digitorum longus. Asterisks indicate significant Δ^15^N differences between two N fractions (P<0.01, independent Student *t*-tests).

All sampled body N fractions were ^15^N enriched compared to the diet, except for the muscle free AA fraction and body urea, which were ^15^N depleted, and urinary urea, which had a δ^15^N similar to that of the diet. The pool-to-diet discrimination values (Δ^15^N_pool_ = δ^15^N_pool_−δ^15^N_diet_) displayed major variations between the different sampled tissues and N fractions, with values ranging from −1.5 to 5‰ (Figure 1). Interestingly, in all the tissues sampled, P fractions had higher Δ^15^N values than non-P fractions, with differences that varied from tissue to tissue: they were for instance larger in muscle and heart and smaller in the kidney Δ^15^N_P_−Δ^15^N_AA_ = 4.8‰, 4.1‰ and 0.6‰, respectively).

### Modeling of body N fluxes and isotopic abundances under standard conditions

#### Model structure and formalism

**Figure 2:**
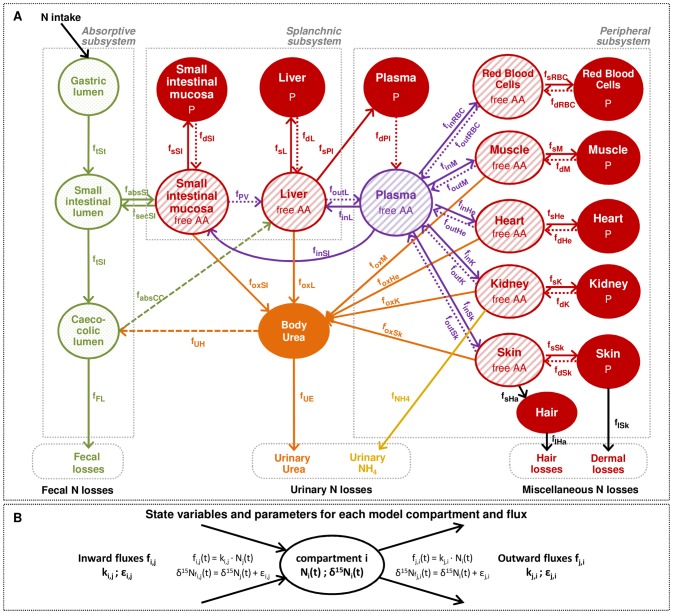
Multi-compartmental model describing the body system of nitrogen fluxes and isotopic signatures in rats. (A) Circles indicate model compartments, representing kinetically homogeneous nitrogen (N) pools and belonging to three subsystems: the absorptive (luminal N contents of the gastrointestinal tract), splanchnic (protein (P) and free amino acid (AA) fractions of small intestine and liver) and peripheral (P and AA fractions of plasma, muscles, kidneys, heart, red blood cells (RBC), skin and hair) subsystems. Arrows between compartments indicate N fluxes corresponding to N transfers and/or metabolic processes. The following fluxes are represented: gastrointestinal N exchanges (gastric emptying, f_tSt_; intestinal transit, f_tSI_; intestinal absorption, f_absSI_; intestinal secretion of endogenous proteins, f_secSI_; caeco-colonic absorption, f_absCC_; body urea transfer toward the colon, f_UH_), N excretion (fecal losses, f_FL_; urinary urea excretion, f_UE_; urinary ammonia production and excretion, f_NH4_; hair losses, f_lHa_; desquamation losses, f_lSk_), tissue P synthesis (f_sT_, for a given tissue T), tissue P degradation (f_dT_), tissue AA catabolism through oxidation (f_oxT_), AA transfers from plasma to tissues (f_inT_) and from tissues to plasma (f_outT_), and AA transfer from the intestine toward the liver through the portal vein (f_PV_). Each oxidation flux f_oxT_ aggregates both the transfer of AA from tissue to liver and their subsequent deamination and use for urea synthesis in liver, and the total body urea production is equal to the sum of all tissue oxidation fluxes. f_UH_ represents enterohepatic urea recycling, (i.e., the part of the urea produced that is not excreted in urine but recycled and hydrolysed by the colonic bacteria), leading to the production of ammonia that can be used either for microbial metabolism or salvaged (i.e., reabsorbed and made available for subsequent metabolic purposes). The amount of ammonia arising from urea hydrolysis that is reabsorbed from the colon is accounted for in f_absCC_ together with the absorption of other endogenous and dietary N compounds. (B) Each model compartment i is characterized by two state variables: its N size (N_i_, mmol N.100 g BW^−1^) and its isotopic abundance (δ^15^N_i_, ‰). Each N flux from compartment j to compartment i (f_i,j_) is characterized by two constant parameters: its reaction rate (k_i,j_, d^−1^) and its fractionation factor (ε_i,j_, ‰). k_i,j_ (k_j,i_) corresponds to the fraction of compartment j (i) that is transferred to compartment i (j) per day, and ε_i,j_ (ε_j,i_) represents the isotopic effect associated with flux f_i,j_ (f_j,i_). The k and ε parameters are used respectively to describe flux size (f_i,j_, mmol N.100 g BW^−1^.d^−1^) and isotopic enrichment (δ^15^N_fi,j_, ‰).

The multi-compartmental model was developed to include the principal N metabolic pathways and pools involved in AA and P metabolism, and to reproduce the δ^15^N values that we observed under standard conditions, while keeping the model size (number of compartments and fluxes) and the complexity of its kinetic equations to a minimum, according to the principle of parsimony. Different candidate models were tested on the basis of their ability to reproduce the data, and the final model retained comprised 21 compartments and 49 fluxes of N exchange and/or transformation (Figure 2A).

Each model compartment is characterized by its total N amount and its isotopic abundance (δ^15^N). Fluxes between compartments are defined by their reaction rate (kinetic parameter k) and an associated isotopic fractionation factor (parameter ε), which reflects the relative affinity of the reaction for ^15^N compared to ^14^N (Figure 2B). The dynamic behaviour of the system (i.e., the kinetic evolutions of N and δ^15^N state variables) is described using a system of ordinary differential equations (ODE) (Table S1). Model equations are based on mass and isotope balance principles and assume mass action laws for both N and ^15^N transfers between compartments.

#### Model calibration and identification of fractionating processes

Experimental measurements were obtained for all the model state variables, N and δ^15^N (Table S2). The model parameter values k and ε were identified using these experimental data and values from the literature (see the Material and Methods section for details). The data from the literature, the equations used to determine model fluxes and the estimated values for k parameters are shown in Table S3.

A simulation of the model, with a constant N intake of 10 mmol N·100 g BW·d^−1^ (i.e., the mean dietary intake observed in rats) allowed us to reproduce experimental N measurements in tissues at the steady state. The global functioning of the system and the N flux values that are predicted are consistent with data in the literature. The model reflects distinct tissue P turnover rates, which range from 3%·d^−1^ in RBC to 150%·d^−1^ in the small intestine mucosa (Table S3). The P synthesis fluxes simulated by the model predict that the splanchnic area contributes about 30% to whole body P synthesis (Figure S1), despite higher turnover rates in splanchnic tissues than in peripheral tissues, in line with findings in the rat [18]–[20]. The model also accounts for distinct fluxes of AA inflow (f_in_) and oxidation (f_ox_) between tissues; by construction, these fluxes are proportional to tissue P turnover and size. For every tissue, the AA oxidation flux represents 29% of total AA utilization for P synthesis and urea production (%ox = f_ox_/(f_ox_+f_s_)), whereas the importance of AA exchange fluxes between plasma and tissue (f_in_ and f_out_) varies depending on the tissues concerned. For instance, the AA inflow accounts for 150% of the P synthesis flux in the muscle but for only 80% in the skin (Figure S2), as set from literature values [21]–[25]. In addition, concerning the N fluxes that were not *a priori* fixed, the model was able to predict a urea recycling efficiency of 18% (calculated as the proportion of daily produced urea that is not excreted but rather hydrolysed in the colon, f_UH_/f_UP_; Figure S1). This value is consistent with estimates of urea recycling efficiency in humans [26]–[30] but is higher than the values reported in rats [31], [32].

The δ^15^N differences observed between tissues and N fractions indicate that isotope fractionation indeed occurs in some metabolic pathways, meaning that such pathways preferentially utilize one of the two N isotopes. This isotopic fractionation was represented mathematically in the model through the fractionation factor parameters ε, whose values were estimated in order to reproduce the δ^15^N values observed experimentally in compartments. According to the parsimony principle, only the fractionation factors required to account for the observed δ^15^N differences were estimated at non-null values. By calibrating the model's ε parameters, we thus identified the metabolic pathways that were most likely to be associated with isotopic fractionation, and in this way we detected numerous and distinct fractionating processes (). First, positive fractionation factors associated with tissue P synthesis (ε_s_), with different values depending on tissues, adequately accounted for the observed systematic ^15^N-enrichment in the P fraction over the AA fraction of each tissue. Second, the measured δ^15^N variations between AA fractions in the different tissues were accurately reproduced by integrating non-null fractionation factors associated with the AA oxidation processes (ε_oxT_), without any isotope effect being associated with AA transfer fluxes between tissues and plasma (i.e., ε_inT_ = ε_outT_ = 0). The estimated ε_oxT_ values differed between tissues and were negative for all tissues except for muscle. Third, non-null fractionation factors for intestinal secretion (ε_secSI_), colonic absorption (ε_absCC_), urinary excretion (ε_UE_) and ammonia production (ε_NH4_) were required to reproduce the differences in δ^15^N measured in N excretion pools.

### Simulation of Δ^15^N trajectories in response to simple flux perturbations (Simulations #1 & #2)

**Figure 3:**
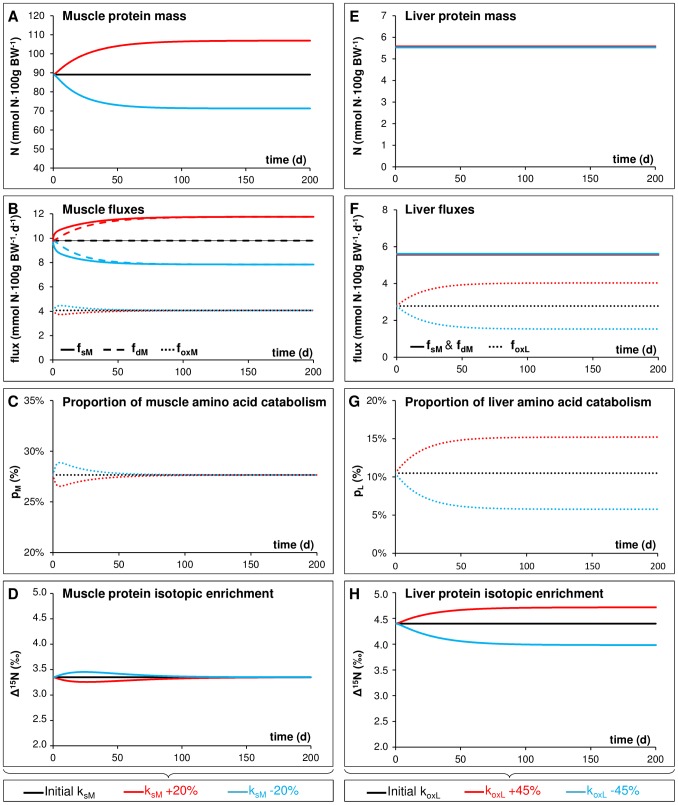
Predicted changes in protein masses, fluxes and isotopic abundances in muscle and liver following simulated variations of the nitrogen fluxes in these tissues (Simulations #1 and #2). Model predictions of dynamic changes in protein mass, nitrogen fluxes and protein-to-diet ^15^N enrichment (Δ^15^N) in muscle (Simulation #1, A–D) and in liver (Simulation #2, E–H) were obtained by simulating two typical kinds of nutritionally-induced changes in the nitrogen fluxes of these tissues, i.e., variations in muscle protein turnover and hepatic amino acid catabolism, respectively. Under Simulation #1 (A–D), reported elemental and isotopic variations resulted from a simulated and gradual 20% increase or decrease in the muscle protein synthesis rate (k_sM_) with consequent changes in muscle protein mass. Under Simulation #2 (E–H), reported variations were obtained from a simulated and gradual 45% increase or decrease in the hepatic amino acid oxidation rate (k_oxL_) without any impact on the liver protein mass as a result of a concomitant counterbalancing modulation in urea recycling (through an increase in k_UH_ and k_absCC_). The p index represents the relative proportion of amino acids entering the tissue that is directed toward catabolism by comparison with net protein synthesis (p_T_ = f_oxT_/(f_outT_+f_oxT_+f_sT_−f_dT_), with T corresponding to muscle (p_M_, panel C) or liver (p_L_, panel G)).

Using the calibrated model, we performed model simulations to identify which flux modulations could explain and induce the Δ^15^N variations observed under specific nutritional or physiological conditions, and also conversely to determine which information could be inferred from Δ^15^N variations relative to underlying modifications in N metabolism. We assumed that ε factors were intrinsic parameters of the metabolic pathways so that they would not be impacted by changes in N fluxes induced by changes in conditions. Therefore, in this simulation phase, we only modified the values of the parameters k, to study the consequences of N flux value changes on tissue Δ^15^N. Model simulations were performed while changing the model fluxes either individually (as reported in this section) or under certain specific combinations (as reported in the next section), so as to mimic the principal types of probable flux modulations occurring in response to changes in the physiological or nutritional conditions (see the Material and Methods section for details on these model simulations). We first investigated the effect of individual changes in each of the main classes of N fluxes (i.e., tissue P turnover, tissue AA oxidation or AA transfers between plasma and tissues). We simulated tissue Δ^15^N trajectories in response to these specific changes in cases where the N amounts in compartments either evolved (Simulation #1 with distinct initial and final elemental steady states, Figure 3A–D) or remained constant as a result of flux compensations re-establishing homeostasis (Simulation #2 with similar initial and final elemental steady states, Figure 3E–H).

As a general rule, the model predicted that variations of fluxes located in a catenary structure with bidirectional exchanges (e.g., the bidirectional fluxes of P synthesis and degradation) only transiently affected Δ^15^N values between two successive elemental steady states. More specifically, we predicted that modifications of the P turnover rates in tissues would not change the isotopic signatures of tissue P at steady state. Indeed, asynchronous variations in the tissue P synthesis and degradation fluxes, which impact tissue P size, induced only temporary changes in tissue AA and P Δ^15^N values during the transient phase of tissue P accretion or depletion, with Δ^15^N values returning to their initial levels at the final isotopic steady state (Simulation #1, Figure 3A–D). For instance in muscle, decreasing (or increasing) the P synthesis flux by decreasing (or increasing) the P synthesis capacity (k_sM_) induced a progressive decrease (or increase) in the P pool size and secondarily in the P degradation flux, until a new elemental steady state was reached. The transition between two distinct elemental steady states, during which an imbalance between the P synthesis and degradation fluxes created a P net loss (or gain), was associated with a transient increase (or decrease) in the P Δ^15^N value. Moreover, we predicted the same variations in the P mass and Δ^15^N values with an increase (or decrease) in the P degradation capacity (k_dM_) than with a decrease (or increase) in k_sM_. More generally, the model predicts that in any tissue, a P accretion (or depletion) is associated with a temporary decrease (or increase) in P Δ^15^N. It should be noted that in all cases, the Δ^15^N variations predicted were quantitatively small (Δ^15^N variations of only 0.1‰ for 20% changes in the P mass).

By contrast, variations of fluxes located at a metabolic branchpoint that induced permanent changes in the allocation of fluxes between competitive pathways (e.g., changes in the relative orientation of tissue AA towards oxidation vs. P synthesis, or changes in AA transfers between plasma and tissues) were predicted to result in more marked and, above all, persistent variations in Δ^15^N values, even without any variation in the elemental steady state. For instance, an increase in hepatic AA oxidation (counterbalanced by an increase in urea recycling in order to maintain homeostasis) induced a progressive and lasting increase in hepatic P Δ^15^N, with higher Δ^15^N values at the final than at the initial isotopic steady state (Simulation #2, Figure 3E–H).

Globally, in terms of the flux variations that effectively impact tissue Δ^15^N values, our results can be summarized by considering changes in the tissue p ratio. The p ratio corresponds to an integrated index of the relative metabolic utilization of tissue free AA that we defined as the proportion of AA that are directed toward oxidation rather than used for net P synthesis or exported to the circulation (p = f_ox_/(f_ox_+f_out_+f_s_−f_d_), Figures 3C and G). We indeed demonstrated that differences in the Δ^15^N of a given tissue between two steady states resulted from permanent changes in the p ratio, whereas changes in the bidirectional fluxes of P synthesis and degradation induced only transient changes in the p ratio and did not modify steady-state Δ^15^N values. The significance of p to steady-state Δ^15^N values was also analytically noticeable from the model equations (Table S1). For instance, the Δ^15^N steady-state value in muscle P (Δ^15^N_MP_) depends on only two factors, the Δ^15^N of the plasma AA supplied to muscle (Δ^15^N_PlAA_) and this integrated index p (since Δ^15^N_MP_ = Δ^15^N_PlAA_−p*ε_OX_−ε_S_, with ε_OX_ and ε_S_ being the fractionation factors for AA oxidation and P synthesis, respectively). Furthermore, when a metabolic change in a given tissue modifies its own P and AA Δ^15^N values, outward transport of this tissue AA consequently modifies the plasma AA Δ^15^N value and can thus also secondarily modify the Δ^15^N values of other body tissues, even if there are no associated metabolic changes in these tissues. For instance, after a concomitant increase in splanchnic oxidation and urea recycling, our model predicted that all body tissue Δ^15^N values were changed and that the Δ^15^N trajectories and the time required for tissue P to reach the new isotopic steady state varied between tissues (Figure S3), depending on their specific rates of plasma AA uptake and P turnover (Table S4 for the characteristic times required to reach 50% and 95% of isotopic equilibrium in each tissue).

### Predicting Δ^15^N variations in response to nutritionally-induced changes in N fluxes (Simulations #3 & #4)

A growing body of evidence in the literature suggests that the isotopic signatures of tissues vary according to the quality and quantity of dietary N intake. More specifically, several studies have reported an increase in tissue Δ^15^N with a decrease in dietary P quality (i.e., when the qualitative and/or quantitative supply of AA in dietary P do not match the AA demand of the individual) [8], [13], [33]–[37] and in cases of dietary restriction or fasting [38]–[42]. However, the Δ^15^N variations reported have sometimes been conflicting and difficult to interpret because of differences in the experimental conditions and the presence of confounding factors, such as concomitant changes in both the quality and quantity of P intake or the heterogeneous extent of body mass depletion in the event of starvation. In all cases, the underlying mechanisms proposed for associated isotopic variations remain speculative. Using the calibrated model, we simulated several scenarios consisting in different N flux modifications that might result from moderate (e.g. variation in P quality; Simulation #3) or severe (e.g. fasting conditions; Simulation #4) nutritional modulations.

#### Model-predicted effects on Δ^15^N values of qualitative and quantitative variations in P intake that do not alter the N balance (Simulation #3)

Several studies have shown that reducing dietary P quality increases AA oxidation and urea production [28], [43], [44]. To study the effect of a change in dietary P quality on tissue Δ^15^N values, we performed various model simulation scenarios that consisted in increasing total urea production by increasing all or only some tissue AA oxidation fluxes. Such modifications are equivalent to increasing the proportion of AA directed towards catabolism rather than P synthesis (i.e., increasing p = f_ox_/(f_ox_+f_s_−f_d_+f_out_) through a specific increase in %ox = f_ox_/(f_ox_+f_s_)), or in other words, reducing tissue P synthesis efficiency. Other N fluxes in the system were redistributed to preserve the whole body N balance and the N amount in each compartment, in order to mimic modulations that might occur in the case of a slight decrease in dietary P quality (i.e., with P requirements being still met and with no effect on the N balance and steady state). The model predicted that steady-state Δ^15^N values were affected differently, depending on how and which AA oxidation fluxes were modified. An increase in AA oxidation fluxes in all tissues, or only in peripheral tissues, resulted in lower P muscle Δ^15^N, with little change in the Δ^15^N values of liver and SI P. By contrast, an increase in oxidation fluxes in splanchnic tissues only led to higher Δ^15^N values in all tissues. According to these simulations, it appears that measuring tissue Δ^15^N variations under particular nutritional conditions could be used to identify the origin and extent of an increase in urea production at the whole-body level.

**Figure 4:**
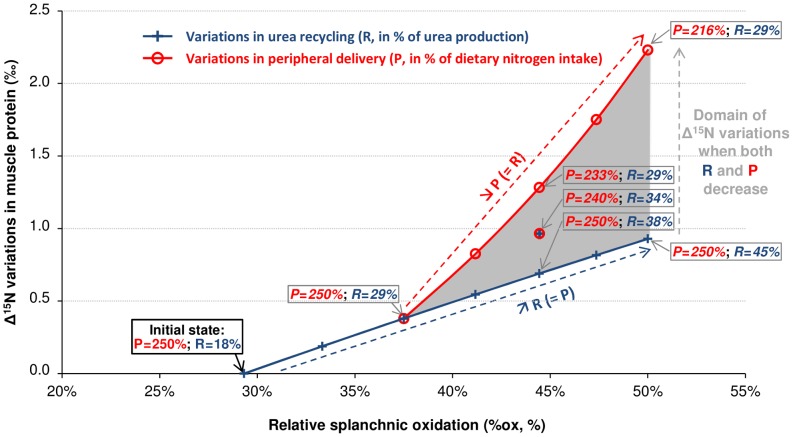
Variations in muscle nitrogen isotopic abundances in relation to combined variations in splanchnic oxidation, urea recycling and peripheral delivery (Simulation #3). Variations in ^15^N enrichment over the diet (Δ^15^N) of muscle proteins were simulated in response to balanced, homeostatic changes in relative splanchnic oxidation (%ox, in %) and in the efficiencies of urea recycling (R, as a % of urea production) and peripheral delivery (P, as a % of dietary nitrogen intake), which might result from qualitative and/or quantitative variations in dietary protein intake. %ox is defined as the proportion of splanchnic amino acid utilization for protein synthesis and oxidation (%ox = f_oxSpl_/(f_oxSpl_+f_sSpl_), with f_oxSpl_ = f_oxL_+f_oxSI_ and f_sSpl_ = f_sL_+f_sPl_+f_sSI_), R = f_UH_/f_UP_ and P = f_outL_ (see Figure 2). Variations in %ox were simulated through changes in the k_oxSI_ and k_oxL_ parameter values, while variations in R and P were respectively achieved through changes in the k_UH_ and k_outL_ parameter values. The blue line corresponds to simulations under which a 0 to 71% increase in the initial %ox (i.e., %ox increasing from 29% to 50%), was entirely offset by a 0% to 148% increase in the initial R (i.e., R increasing from 18% to 45%), with no change in P. The red line corresponds to simulations under which a 28 to 71% increase in the initial %ox was counterbalanced by a decrease in P ranging from 0 to 13% (i.e., P decreasing from 250% to 216%), with R being fixed at 29% (i.e., 60% higher than its initial value). The shaded area between the red and blue lines corresponds to intermediate scenarios under which the increase in %ox was counterbalanced to varying degrees by an increase in R (ranging from 60% to 148%) and a decrease in P (ranging from 0 to 13%). Within this area, a similar Δ^15^N variation (corresponding to a horizontal line) could be obtained for different combinations of R and P variations. Variations in Δ^15^N are expressed as the difference between final and initial steady state Δ^15^N values.

In addition, an increase in the splanchnic AA oxidation and urea production could be balanced in different ways to preserve the whole-body elemental steady state. We compared scenarios under which supplemental urea production was either entirely or only partly offset by a similar increase in urea recycling (i.e., an increase in urea recycling fluxes f_UH_ and f_absCC_ that entirely or only partly balanced an increase in the urea production flux f_UP_) (Simulation #3, Figure 4 and Figure S4). Our model predicted that tissue Δ^15^N values were differently affected depending on how the increase in splanchnic urea production was offset. In the cases where the increase in splanchnic AA oxidation efficiency (30% to 70% increase in %ox) was totally counterbalanced by an increase in urea recycling efficiency (60% to 150% increase in f_UH_/f_UP_), Δ^15^N values were increased by 0.4 to 0.9‰ in all tissues, except in the SI where they rose slightly more moderately by 0.3% to 0.7‰. Tissue Δ^15^N values were more markedly increased (from 0.4% to about 2.4‰) in the cases where a similar increase in splanchnic AA oxidation efficiency was only partly counterbalanced by an increase in urea recycling efficiency (60% increase in f_UH_/f_UP_) but also by a decrease in AA systemic delivery (3% to 13% decrease in f_outL_). In this latter scenario, peripheral metabolism was reduced (with tissue P turnover falling by 12% to 50%), but without any effect on tissue masses. As a general rule, we noticed, from model simulations, that when AA catabolism was increased, the resulting increase in tissue Δ^15^N was even greater as peripheral metabolism was also concomitantly altered. However, according to our simulations, some increases in AA oxidation combined with different degrees of urea recycling efficiency and alterations of peripheral metabolism could result in similar tissue Δ^15^N variations. Yet, importantly, by analysing the Δ^15^N predictions obtained across all these scenarios we were able to show that the predicted Δ^15^N values of fecal losses (but not of urinary urea) were linearly correlated with urea recycling efficiency (Figure S5).

In addition to the above-mentioned qualitative changes, we also tested the effects of quantitative changes in N intake, always in the context of an unchanged N steady state, since urea production and excretion were changed accordingly in order to maintain a null N balance. We predicted that an increased P intake induced similar tissue Δ^15^N variations to a reduced P quality. Tissue Δ^15^N values were indeed found to be higher when the greater urea production induced by an excessive N intake resulted entirely from an increase in splanchnic AA oxidation than from an increase in peripheral AA oxidation.

#### Model-predicted effects on Δ^15^N values of nutritional disruptions of the N balance (Simulation #4)

**Figure 5:**
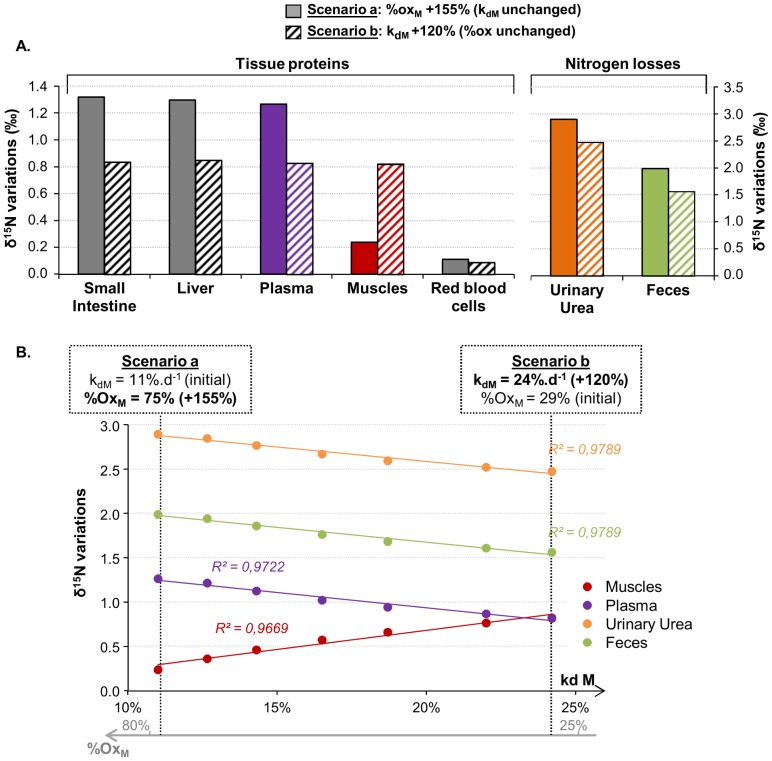
Variations in nitrogen isotopic abundances under starvation depending on associated metabolic changes (Simulation #4). Predicted variations in nitrogen isotopic abundances (δ^15^N) of tissue proteins, urine and faeces were obtained when mimicking starvation conditions, by simulating a zero nitrogen intake for 7 d and altered muscle nitrogen fluxes causing a loss of muscle protein mass. We compared several scenarios under which an identical 45% decrease in muscle protein mass was obtained by increasing, to varying degrees, the muscle protein breakdown rate (k_dM_, corresponding to the protein degradation efficiency) and/or the proportion of muscle amino acid oxidized rather than utilized for protein synthesis (%ox_M_ = f_oxM/_(f_oxM_+f_sM_), see Figure 2). (A) Scenarios a and b represent the two extreme scenarios. Scenario a corresponds to a 155% increase in %ox_M_ (%ox_M_ = 75% vs 29% before starvation, through an 80% increase in k_oxM_ and a 75% decrease in k_sM_) without changing k_dM._ Under scenario b, k_dM_ is increased by 120% (k_dM_ = 24%.d^−1^ vs 11%.d^−1^ before starvation) without changing %ox_M_. (B) δ^15^N variations were predicted in some characteristic pools (muscle and plasma proteins and urinary urea and fecal nitrogen) for intermediary combinations of changes in both %ox_M_ (from 0 to 155% of its pre-starvation value) and k_dM_ (from 0 to 120% of its pre-starvation value) leading to the same muscle protein mass loss: δ^15^N variations in pools are linearly correlated with changes in k_dM_ and %ox_M_ (correlation coefficients R^2^ ranging from 0.97 to 0.98, *P<0.01*).

In this section we present the effects of more extreme changes in N fluxes that lead to a disruption of the body N balance. In particular, we investigated the extreme case of starvation by setting the simulated N intake to zero (Simulation #4, Figure 5). It should be noted that, contrary to previous simulations under which compartment sizes remained constant, in this case the amount of N in compartments decreased as a consequence of the shortage in N input, and the system did not reach an elemental or isotopic steady state.

It is known that after a few days of starvation, P degradation is increased and AA oxidation becomes a major contributor to body energy production [45]–[47]. A higher level of P catabolism is particularly observed in muscle, leading to a major loss of muscle P mass [46]. In our model, under the conditions of a null N intake, muscle P losses of similar amplitude could be achieved by modulating different fluxes. We compared scenarios under which a similar 45% reduction in muscle P mass was obtained after 7 days of starvation, either by increasing the muscle P breakdown rate (k_dM_, scenario b in Figure 5) or by decreasing muscle P synthesis efficiency (i.e., decreasing the muscle P synthesis rate k_sM_ and increasing AA oxidation capacity k_oxM_, scenario a in Figure 5) or by combinations of these two mechanisms. Under these simulated scenarios, intestine and liver P masses fell by 55% and 35%, respectively. The model predictions showed that P mass loss was consistently associated with higher δ^15^N values in all tissues, but with amplitudes that differed depending on the simulated scenario. An increase in the muscle P breakdown flux alone caused a similar increase in all tissue δ^15^N values, whereas a decrease in muscle P synthesis efficiency alone led to a greater δ^15^N increase in splanchnic tissues but smaller δ^15^N increase in muscle (Figure 5A). Under our different scenarios, which consisted of intermediary combinations of decreases in k_sM_ and increases in k_dM_, the predicted δ^15^N variations in tissues were linearly correlated with the muscle breakdown rate, with negative correlations in splanchnic tissues and a positive correlation in muscle (Figure 5B). Muscle was the only tissue in which the starvation-induced δ^15^N increase was greater when the protein loss resulted more from an altered P breakdown and less from altered AA oxidation.

## Discussion

### Understanding the isotopic trophic shift and inter-tissue Δ^15^N variations by identifying the metabolic processes that generate isotopic fractionation

Our experimental data, as well as the model predictions for tissue Δ^15^N values, are consistent with the few findings available in the literature on rodents [13], [33], [34], [48]–[52] (Table S5). In contrast, whereas in the literature urine is usually reported to be ^15^N-depleted compared to the diet [13]–[15], [53]–[59], in this study we observed and simulated Δ^15^N values in urine that did not differ significantly from zero. Given that urinary N accounts for the largest part of N losses, the isotopic composition of N losses was globally similar to that of N intake (i.e., null Δ^15^N in N losses). As a result, amounts of ^15^N intake and losses were equal, which ensured that the body was globally at isotopic steady state at the time of the measurements. A negative Δ^15^N in N losses has often been put forward to explain the trophic shift (i.e., positive Δ^15^N in body tissues), but this would mean a permanent and endless Δ^15^N increase in adult body tissues. Because no net gain of ^15^N or net loss of ^14^N occurs in an adult animal at isotopic steady state, the trophic shift probably result from ^15^N accumulation during the growth period before adulthood, when animals are in positive N balance. Since N deposited in tissues is ^15^N-enriched relative to excreted N, net gain of N in tissues during growth should be associated with preferential ^15^N bioaccumulation. In addition, the higher ^15^N abundance observed in P than in AA fractions of tissues is a novel and important finding that had not previously been reported, because tissues are usually analysed as a whole, without making a distinction between the AA and P fractions. It should be noted that some other N-containing compounds like nucleotides in tissues, creatine in muscle and uric acid in plasma are probably present, but in relatively small concentrations, in what we considered as the “free AA” fractions, since there is no way to separate these minor compounds from the main free AA. However, based on their known relative concentrations, these potential contaminants may not alter significantly our estimates of the free AA isotopic values.

As suggested by *in vitro* studies [9], [60]–[64], our model confirmed that some metabolic pathways are associated with isotope effects that explain the well-known, but still poorly understood, δ^15^N trophic step, and also the observed, but also misunderstood, Δ^15^N differences between and within tissues. Whereas there are very few quantitative data in the literature concerning the amplitude of the isotope fractionation associated with metabolic pathways *in vivo*, one highlight of our work is that it enabled the localisation and quantification of these isotope effects (Table S3, ε values). Globally, according to our model, no net isotope effects were likely to be associated with pathways involving simple N transport without metabolic transformation (e.g., AA exchange fluxes between plasma and tissues, N transfers throughout the gastrointestinal tract and N losses via the faeces, hair and desquamation, etc.), inasmuch as these isotope effects were not required to reproduce intra-individual Δ^15^N variations. Conversely, we identified the existence of isotope effects associated with the tissue AA oxidation and urea production pathways. These predictions are in line with the fact that the most likely processes inducing isotopic fractionation are those which involve enzymatic transfers of amino groups such as deamination and transamination reactions [9], [11], [17]. However, although we were able to confirm the existence of an isotope effect associated with deamination, we showed that this mechanism alone could clearly not explain the δ^15^N trophic shift in tissues. Simple whole-body models, which do not distinguish between different body tissues and consider all body P as a single compartment, conclude that the isotopic fractionation associated with AA catabolism and N elimination is sufficient to explain ^15^N accumulation in tissues [11], [12], [16], [65]. But thanks to the multi-tissue representation of our model, we were able to demonstrate that isotope effects with distinctive amplitudes are necessarily involved in several metabolic pathways to give rise to the Δ^15^N variations observed among tissues and among N fractions within tissues. More specifically, we predicted varied non-null ε values associated with urea production and urinary excretion fluxes, as well as with P synthesis fluxes in tissues and some intestinal secretion and reabsorption fluxes. It has to be noted that the non-null ε factors in our model do not strictly represent isotope effects associated to specific individual chemical processes, but rather aggregate several fractionation processes that may occur on different pathways. Generally speaking, non-null ε factors can reflect the heterogeneity of the precursor pool and the fact that only a subset of the compounds in the precursor pool (some specific AA for instance), with a distinct δ^15^N, is used in a given pathway. In the case of exchanges that involve opposite reactions (e.g., intestinal absorption and secretion, or P synthesis and breakdown), non-null ε factors usually represent the net fractionation effect associated with the bidirectional exchange (see Text S1 for a further discussion on the physiological plausibility of our estimated ε values). To avoid over-parameterization of the model, we also chose to represent all its N fluxes by linear, first-order dynamics. Indeed, on grounds of parsimony, it was not necessary to make the model equations nonlinear and more complex (e.g., using saturable transfer laws), since the modeling results were judged satisfactory with regard to the general knowledge on the functioning of the N metabolic system and our experimental data.

### Identifying the ability of Δ^15^N values to sign the metabolic impact of changes in nutritional conditions

From the model simulations, we observed that nutritionally-induced variations in P turnover and AA catabolism fluxes led to Δ^15^N changes that could be of varied extents and durations (transient or permanent). Moreover, when comparing the initial and final steady states, we found that changes in the sizes and Δ^15^N of body N pools were not systematically coupled: compartment sizes may differ between two steady states without associated differences in Δ^15^N values, and *vice versa*. For instance, in the case of net P accretion or depletion, Δ^15^N values were similar at the initial and final steady states while the elemental steady states were different. Δ^15^N were only temporary changed during the period necessary to achieve the new steady state (i.e., when the P synthesis and degradation fluxes were unbalanced). More specifically, a permanent P accretion (or inversely, depletion) was associated with a transient decrease (or increase) in the tissue P Δ^15^N value (Figures 3A–D). Because, according to our predictions, the isotopic variations induced by alterations to P synthesis and degradation fluxes are only transient and of small amplitude, they will probably be very difficult to detect in practice. Therefore such fluxes modulations are neither sufficient nor necessary to explain Δ^15^N differences that can be observed between two distinct metabolic states. The fact that tissue Δ^15^N values do not record the entire history of P anabolic and catabolic phases that specifically result from transient imbalances between P synthesis and degradation probably limits the degree of both the inter- and intra-individual Δ^15^N variability in a given tissue. In contrast to the limited impact of modulations in bidirectional fluxes, variations in fluxes involved in metabolic branched pathways, such as modifications of the relative orientation of tissue AA toward their different metabolic and transfer pathways, are predicted to lead to lasting changes in Δ^15^N values (i.e., with distinct final and initial isotopic steady states), even without a concomitant variation in the elemental steady state (Figures 3E–H). Generally speaking, based on both our analytical (model equations, in Table S1) and numerical (model simulations, Figures 3–4) results, we can predict that the steady-state isotopic signature of a given tissue should depend both directly on its own metabolism and indirectly on the isotopic signatures of other tissues. Indeed, in peripheral tissues such as muscle, variations in metabolism would directly affect tissue AA and P Δ^15^N values through changes in the proportion of AA catabolism relative to net P synthesis and outward transport (as represented by the integrated index p). Besides, because of blood-tissue AA exchanges, a metabolic-induced Δ^15^N change in a given tissue could modify plasma Δ^15^N values, and thus also secondarily those of other body tissues without the metabolism of the latter necessarily being changed. In splanchnic tissues, which have a more central and connected position in the metabolic network, steady-state Δ^15^N values are predicted to be affected by local tissue metabolism and also by the relative contribution of other metabolic pathways, such as urea recycling and salvage by N intestinal reabsorption (see Table S1 for detailed equations). Furthermore, when simulating Δ^15^N trajectories in different body tissues after a metabolic change specifically affecting splanchnic tissues, we predicted that Δ^15^N values varied in all tissues but according to different kinetics, the new isotopic steady states being reached at different speeds (Figure S3). These results are of particular interest in terms of determining the time required for such metabolic alterations to become isotopically detectable, as a function of the tissue under study. This kinetic characteristic also paves the way to using isotopic measurements obtained concomitantly in several tissues to estimate the timing of a metabolic alteration, or, when used in ecological studies, to infer the time of a diet-shift or migratory movement in the context of dietary δ^15^N changes [17], [66]. For such ecological applications, the predicted half-lives and times needed for tissues to reach their isotopic steady state after a dietary and/or a metabolic change are of considerable value (Table S4).

We also used the model to investigate how tissue Δ^15^N values might vary under particular changes to dietary P intake and which N flux alterations would most likely be responsible for reported Δ^15^N variations under such conditions. Globally, under moderate qualitative or quantitative changes in the dietary P intake, which preserve the whole body N balance, our simulations suggested that Δ^15^N would be minimal when the P intake optimally matched AA demand and N requirements. Indeed, we predicted higher tissue Δ^15^N values when the N intake deviated qualitatively or quantitatively from an optimum intake. This results from the fact that an altered dietary P quality, due to a less well-balanced AA composition, or an excessive P intake, leads to a greater relative orientation of tissue AA to the catabolic vs. anabolic pathways, and consequently to less efficient tissue P synthesis and dietary P anabolic use. These predictions agree with the results of studies performed in several different species that have shown that Δ^15^N decreased as the dietary P biological value or anabolic use efficiency increased [36], [67], [68].

More specifically, we simulated different N flux modulation scenarios that could result from a change to dietary P quality. These scenarios consisted mainly in increasing tissue AA oxidation fluxes (i.e., decreasing tissue P synthesis efficiency) in different tissues and to various extents. The model predicts that an increase in AA catabolism in splanchnic or peripheral tissues would result in contrasting Δ^15^N changes (increase or decrease, respectively) in the tissue P. Since published studies have mostly reported higher Δ^15^N values in tissues with dietary P of poorer quality [8], [13], [33], [34], [68], our simulation results suggest that the higher urea production induced by a P of poorer quality is caused by an increase in the oxidation of splanchnic rather than peripheral AA. The model also predicts that the amplitude of increase in tissue Δ^15^N values after an increase in splanchnic AA oxidation would be all the larger as the resulting increase in urea production is not entirely balanced by an increase in urea recycling but, rather, associated with an alteration to peripheral metabolism (Figure 4). However, tissue Δ^15^N values would probably not constitute specific markers of the relative changes in urea recycling and whole peripheral metabolism, because similar Δ^15^N values could be obtained with distinct sets of urea recycling efficiency and peripheral AA delivery values. In addition, the Δ^15^N variations predicted in urinary and body urea result from numerous and possibly opposing isotopic variations in the splanchnic and peripheral tissues, so that Δ^15^N variations in urinary and body urea at an isotopic steady state are likely not to be informative about body N flux modulations. In contrast, faeces Δ^15^N might constitute an interesting marker of enterohepatic urea recycling efficiency (Figure S5). Globally, the simulations demonstrate that combined measurements of Δ^15^N changes in various tissues and N pools might help to disentangle the underlying intricate metabolic modulations associated with changes in dietary P quality.

Our model also provides insight into isotopic variations under more stringent nutritional alterations to metabolic fluxes, such as during nutritional stress, fasting or starvation. Although muscle P wasting has been well described under prolonged starvation, the flux modulations involved have not been fully identified [69]. Our simulations showed that a drastically reduced or zeroed N intake could induce a global δ^15^N increase in most body and elimination pools. Amplitudes of the simulated δ^15^N variations differed between tissues and depended on the N flux modulations responsible for P mass loss (Figure 5). The predicted δ^15^N increases in urine and most tissues (except red blood cells) in response to starvation are in line with most literature values reported for mammals experiencing N imbalance [14], [15], [40], [42], although conflicting results have been reported across various tissues, species and conditions of nutritional restriction [5], [6], [29], [30]. For instance, some studies showed a general increase in the δ^15^N values of various tissues in fasting or P deprived birds [29], [30], whereas other studies reported δ^15^N changes in only some tissues (e.g., in liver, SI mucosa and heart, but not in muscle, in fasting Arctic ground squirrels during hibernation [40]) or no δ^15^N changes in tissues despite an increase in excreta δ^15^N [45], [46]. Our model was able to reproduce qualitatively such different results and our simulations suggest that δ^15^N variations under starvation may be different depending on how and which metabolic fluxes are altered. We indeed found that a muscle mass loss that is mainly induced by an increase in the muscle P degradation, should lead to a similar increase in the δ^15^N values in all body tissues, which is consistent with reports on quails under starvation [39]. By contrast, when muscle mass loss is caused by an alteration to muscle P synthesis, together with an enhanced oxidative utilization of muscle AA, we predicted a greater δ^15^N increase in splanchnic tissues and almost no δ^15^N variations in muscle, as has been reported in hibernating squirrels [40]. A global body δ^15^N increase in response to starvation had also been predicted by previous simple models in the literature [11], [16], which considered all body P as a unique and homogeneous compartment. In such models, urinary N excretion was considered as the only fractionating process, with a preference for ^14^N, so that δ^15^N in the single-body P pool systematically increased in line with an increase in the proportion of excreted vs. ingested N. Unlike these over-simplified models, our multi-tissue model takes into account possible isotopic fractionating processes associated with between and within tissue N fluxes, thus explaining our seemingly divergent experimental results. Indeed, our model can explain the tissue-specific δ^15^N variations reported during starvation as resulting not only from enhanced urinary excretion but also possibly from a tissue-specific increase in endogenous P degradation, leading to an enhanced re-use of ^15^N-enriched AA for P synthesis after their inter-organ redistribution. More generally, in situations of N imbalance, our model is able to discriminate between variations in the P synthesis and breakdown fluxes leading to similar P mass variations.

### Conclusions

Our simulation results thus demonstrate the physiological plausibility of the model we have developed. The behaviour of the model is in line with general knowledge on the functioning of the N metabolic system and with the fragmented data that have been reported on nutritionally-induced Δ^15^N variations. Thanks to its multi-tissue representation, our model provides a detailed and integrated insight into the partitioning of different N metabolic fluxes between and within tissues, and a clearer understanding of the metabolic processes that generate isotopic fractionation and their interactions. We have shown that, contrary to the common hypothesis, urea production is not the only process responsible for the well-known, but poorly-understood, δ^15^N trophic shift (i.e., positive Δ^15^N values in tissues). Numerous other metabolic processes, such as P synthesis, AA intracellular metabolism, intestinal absorption and urea recycling through the colon, are actually likely involved in the accumulation of ^15^N in tissues. Interestingly, our findings suggest that tissue Δ^15^N could be used as an indicator of how well the diet matches the metabolic demand for AA in tissues. The existence of such a natural isotopic biomarker paves the way towards better assessing the notion of dietary P quality under various physiopathological conditions. In addition, using model simulations, we were able to highlight that tissue Δ ^15^N values are closely related to the distribution of N fluxes within the body, and that Δ^15^N measurements could therefore be used as biomarkers for the metabolic impact of nutritional conditions. Although in a given tissue Δ^15^N often seems to be a sensitive but rarely specific marker of particular dietary and metabolic conditions, we showed that simultaneous measurements of Δ^15^N in various tissues can be used to characterize a particular metabolic state. Accordingly, the present study thus constitutes proof of concept that natural N isotope abundances are interpretable biomarkers for the metabolic impact of nutritional conditions. A multi-tissue mechanistic modeling approach, such as that developed during this study, in order to understand the mechanisms underlying natural isotopic signatures, is a prerequisite for further research on their use in nutrition and physiopathology, in addition to their usual applications in ecology and anthropology [5]. Isotopic signatures at natural levels of abundance therefore appear to constitute a novel and promising tool to investigate how various N fluxes may be reorganised or altered in a coordinated manner to adapt to specific nutritional or physiopathological conditions. They offer interesting applications for the simple and early detection of such conditions and evaluation of the impact of nutritional strategies.

## Methods

### Collection of experimental data

#### Ethics statement

All experiments were carried out in accordance with the recommendations of the NIH Guide for the Care and Use of Laboratory Animals. The study protocol was approved by the Ethics Committee for Animal Experiments (COMETHEA) of the Jouy-en-Josas INRA Centre and AgroParisTech (approval number 11/017).

#### Animals

Ten male Wistar rats, initially weighing approximately 206±9 g, were maintained for 10 weeks under standard conditions and fed with a diet containing 20% of energy as milk P (casein), 55% as lipids and 25% as carbohydrates. The rats were housed individually in a temperature-regulated (22±2°C) room under a 12-h light-dark cycle (dark period from 17:00 to 5:00) and were given free access to food and water throughout the day. After 10 weeks, all the rats, weighing approximately 410±35 g, were euthanized after an overnight fast. The animals were anaesthetised by the inhalation of isoflurane and then killed by rupture of the aorta under deep anaesthesia. Their intestinal contents (in the caecum and colon), urine (in the bladder), blood (plasma and red blood cells) and various tissues (small intestine mucosa, liver, kidneys, tibialis anterior muscle, extensor digitorum longus muscle, heart, skin and hair) were collected. The sampling procedures were carried out as described previously [70]. Briefly, the P and non-P (containing mostly AA) fractions were isolated in each sampled tissue by acid precipitation and the soluble non-P fraction was filtered using 3 KDa cut-off filters to isolate the AA fraction. The urea and AA fractions in plasma, and the ammonia and urea fractions in urine, were extracted using cation-exchange resins. The analytical procedures used to isolate these N fractions were the same as those described previously [70].

#### Elemental analysis and isotopic determinations

The stable isotope ratios of N were determined in each of the N fractions isolated from tissues (P and AA fractions), plasma (P, AA and urea fractions) and urine (ammonia and urea fractions) using an isotope-ratio mass spectrometer (Isoprime, VG Instruments, Manchester, UK) coupled to an elemental analyser (EA 3000, Eurovector, Italy). Internal standards (Tyrosine) were included in every run to correct for possible variations in the raw values determined by the mass spectrometer. Typical replicate measurement errors for these reference materials were ±0.1‰. Results were expressed using the delta notation according to the following equation:
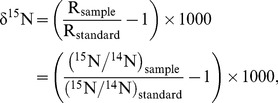
(1)where R_sample_ and R_standard_ are the N isotope ratio between the heavier isotope and the lighter isotope (^15^N/^14^N) for the sample being analysed and the internationally defined standard (atmospheric N_2_, R_standard_ = 0.0036765), respectively, and δ is the delta notation in parts per 1000 (‰) relative to the standard.

The N percentages in each P and AA fraction of the samples were determined using the elemental analyser, with atropine as the standard. Urea concentrations in plasma were determined with a commercial kit using an enzymatic method (Urea kit S-1000, Biomerieux, Craponne, France). Calculations to quantify total N amounts in the various N fractions of tissues, plasma and urine that corresponded to the model compartments are described in detail in Text S2.

Table S2 summarizes all the experimental data used for model calibration.

### Mathematical modeling for data analysis and predictive simulations

#### Model formulation and construction: determination of the model structure and equations

We chose to depict whole-body N metabolism using a multi-compartmental model, with the compartments representing kinetically homogeneous N fractions (i.e., amounts of N in a particular metabolic form (e.g., P, AA, urea, etc.) and at a particular site (e.g., in the intestinal lumen, liver, muscles, etc.), linked by N exchange pathways (i.e., N transfer and/or metabolic transformation). In our mathematical representation, each compartment i was defined by two state variables: its size (i.e., its total N amount N_i_) and its N isotopic abundance (δ^15^N_i_); and each N flux from compartment j to compartment i was characterized by two constant parameters: its rate (k_i,j_) and fractionation factor (ε_i,j_) (Figure 2B). The global dynamic behaviour of the system was thus characterized by two sets of differential equations (details on the derivation of differential equations can be found in Text S3, and differential equations for liver and muscle are given in Table S1).

The first set of differential equations describes the kinetic evolution of the N amount in the model compartments according to the mass conservation principle and assuming that the N fluxes between compartments follow mass action laws. For each compartment i of the model, which is connected to several compartments j, the evolution of its total N amount (Ni) is thus described using an equation with the following form:
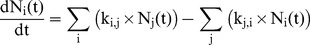
(2)where the k_i,j_ (respectively k_j,i_) parameters are the reaction rate constants of the corresponding N fluxes f_i,j_ from compartment j to compartment i (respectively f_j,i_ from compartment i to compartment j).

The second set of differential equations describes the kinetic evolution of the isotopic abundance of the model compartments, with the flowing general form for compartment i:
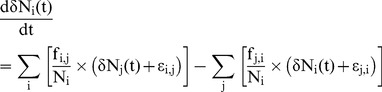
(3)where the ε_i,j_ and ε_j,i_ parameters are the fractionation factors associated with the corresponding N fluxes f_i,j_ and f_j,i_, respectively. These equations are derived by applying the mass conservation principle to the heavy N isotope (^15^N), assuming that the ^15^N fluxes follow the kinetic mass action law, similar to total N fluxes. The isotopic fractionation factors ε account for the potential isotope effects associated with the corresponding metabolic reactions, i.e., for the fact that some metabolic pathways are sensitive to isotope masses so that one N isotope (usually the lighter one) reacts more rapidly than the other (heavier) one, and the ^15^N abundance of the instantaneous flux coming from a compartment differs from that of this source compartment. In our representation, in line with the classical formalism [71], the ^15^N abundance of the instantaneous flux f_j,i_ (δf_j,i_) coming from compartment i is defined by the ^15^N abundance of compartment i and the fractionation factor ε_j,i_ according to the following equation: δf_j,i_ = δN_i_+ε_j,i_. Consequently, a negative ε value reflects a higher affinity of the metabolic pathway for the lighter isotope (i.e., a preferential transfer of ^14^N rather than ^15^N), whereas a positive ε value indicates a preferential transfer of the heavier isotope.

The model was implemented using MATLAB's SimBiology toolbox (version 2.2) and, for the time-course simulations of N and δ^15^N state variables, the ODE system was solved using the MATLAB stiff solver ode15s. The complete set of ODEs describing the system can be found in Table S1.

#### Model calibration under standard conditions: identification of the numerical values for model parameters

Model calibration consisted in estimating the numerical values of the k and ε parameters using the experimental N and δ^15^N values, which were considered to correspond to the values of the model state variables obtained at the elemental and isotopic steady states (i.e., when N amounts and δ^15^N in compartments had reached a constant level). The whole model was calibrated following two successive steps: the k parameters were first defined from the N data and then the ε parameters were estimated from the δ^15^N data, with the k parameters being fixed at their previously determined values.

During the first step, the k parameters were estimated so that, for each model compartment, the N amount predicted by the model at the elemental steady state matched the experimental measurements. For this purpose, the differential equations describing the evolutions of N amounts in compartments (Text S3, Eq. E6) were simplified under the assumption of the elemental steady state (i.e., with the time derivative set at zero). Parameter values were assessed by solving this system of mass balance equations and using the experimentally measured N amounts. Because of the large number of N transfers and the reversibility of most of them, parameters could not be uniquely identified based solely on the experimental data, and the values of some N fluxes were fixed at values obtained from the literature. For instance, for each tissue T (muscle, skin, RBC, kidney, heart), P synthesis and degradation fluxes were determined based on literature values for P turnover rates according to the following equation: f_sT_ = f_dT_ = FSR_T_×T_P_, where f_sT_ and f_dT_ are the P synthesis and degradation fluxes (mmol N·100 g BW·d^−1^) of tissue T, T_P_ is the size of the tissue P compartment (mmol N·100 g BW^−1^), and FSR_T_ the P turnover rate (d^−1^), which differs for each tissue. AA oxidation fluxes (f_oxT_) and inward AA transport fluxes from plasma to tissue (f_inT_) were considered to be proportional to the tissue P synthesis flux as follows: f_inT_ = TI_T_×f_sT_ and f_oxT_ = [%ox_T_/(1−%ox_T_)]×f_sT_, where TI represents the transport index and %ox_T_ the proportion of tissue AA catabolized through oxidation rather than used for P synthesis (%ox_T_ = f_oxT_/(f_oxT_+f_sT_)). According to data in the literature [20]–[24], [72], TI was set at 1.5 for all tissues except the skin, for which it was set at 0.8 [25]. %ox was assumed to be identical in each tissue and calculated so that the total daily urea production equalled the daily N intake, according to mean values in the literature [29], [30], [73]–[75]. The full list of the assumptions and data from the literature used to calibrate the model fluxes is given in Table S3.

During the second phase of the calibration process, the ε parameters were estimated in order to obtain the best fits between the experimental δ^15^N measurements and the δ^15^N model predictions at an isotopic steady state. In the same way as for k parameters, the ε parameters could not be uniquely identified from the experimental data. To solve this identifiability problem, and according to the principle of parsimony, we selected the minimal set of ε parameters that could account for the δ^15^N experimental data, while setting at zero the other fractionation factors that were not necessary to account for the δ^15^N differences observed between tissues and fractions. For instance, the δ^15^N difference observed within each tissue between the AA and P fractions was adequately reproduced by considering a fractionation factor associated with either P synthesis or P degradation, so that we arbitrarily chose to set the fractionation factor associated with P degradation at 0 (ε_d_ = 0) and estimated the isotopic fractionation factor on the P synthesis pathway as the δ^15^N difference between the P and AA fractions for each tissue.

Several model structures that integrated different numbers of compartments and fluxes were tested. For each given structure, the numerical values of the model parameters were estimated by adjusting the model predictions to the experimental and literature data, as detailed above. The model was modified iteratively by changing its structure and then re-estimating the parameter values, until it yielded an adequate fit to the available experimental data. The final model selected is presented in Figure 2, and the final values estimated for the k and ε parameters are given in Table S3.

#### Model simulations to explore the impact of nutritionally-induced metabolic changes on δ^15^N values

Once the model had been calibrated, we performed a sensitivity analysis to better understand the system's behaviour and study the impact of some specific N flux modulations on the δ^15^N values of the various N pools, in order to identify possible isotopic signatures of these metabolic variations. For this purpose, we modified the values of some N fluxes by changing the values of the corresponding rate parameters k, while the ε parameters were kept at their previously estimated values (because these ε parameters represent the absolute isotope effects of the metabolic pathways and consequently should not depend of their rate and flux values). The system was initially in an elemental and isotopic steady state, meaning that N amounts and Δ^15^N values in the different compartments were constant. As a consequence of changes in some N fluxes (with or without variations in the N amount within compartments), the δ^15^N of some compartments deviated from their initial isotopic steady state and evolved over time until they once again reached an isotopic steady state that was either identical or different from their initial one.

We first of all investigated the dynamic changes of body δ^15^N (and more particularly, tissue Δ^15^N trajectories) between their initial and new isotopic steady states in response to changes in some specific tissue fluxes (f_sM_ and f_dM_, or f_oxL_, the muscle P synthesis and breakdown fluxes or the liver AA oxidation flux, respectively) that either modified (Simulation #1) or maintained (Simulation #2) the elemental steady state. To mimic a progressive metabolic adaptation to new dietary conditions, we simulated gradual exponential variations in the constant rates of f_sM_ (k_sM_, Simulation #1) or f_oxL_ (k_oxL_, Simulation #2), so as to obtain final variations of 20% for f_sM_ and 45% for f_oxL_. Under simulation #1, changes in f_sM_ impacted the muscle P mass, so the final and initial elemental steady states were different. Under simulation #2, the initial elemental steady state was maintained by balancing the change in hepatic urea production (i.e., the 45% change in k_oxL_) with concomitant and adequate changes in urea recycling and intestinal N reabsorption (69% and 54% changes in k_UH_ and k_absCC_, respectively). In addition, in order to compare the Δ^15^N trajectories in different tissues after a given metabolic change, we simulated the Δ^15^N kinetics in different tissue P after an instantaneous 45% increase in splanchnic AA oxidation (i.e., with a 45% increase in both k_oxSI_ and k_oxL_ and a sufficient increase in k_UH_ and k_absCC_ to maintain the elemental steady state). We then simulated more complex scenarios that involved combined changes in several metabolic fluxes in different splanchnic and peripheral tissues, designed to mimic altered nutritional and physiological conditions such as a reduction in dietary P quality (Simulation #3) or a short period of starvation (Simulation #4). We chose scenarios that corresponded to nutritional modulations in response to which Δ^15^N variations had been reported in the literature and could therefore be compared to our model predictions. Under Simulation #3, the elemental steady state was preserved through flux redistribution, and we studied the effects of flux variations on the Δ^15^N values at isotopic steady states. By contrast, an elemental steady state was not preserved under Simulation #4, where the N balance became rapidly negative, and with such a scenario of sustained starvation, body compartments could not reach an isotopic steady state: in this case, we reported and analysed the isotopic changes predicted in the body after 7 days in terms of δ^15^N rather than Δ^15^N variations, since the dietary input was zero.

## Supporting Information

Figure S1 Quantitative distribution of the main body nitrogen fluxes in the model calibrated under standard conditions. (TIF) Nitrogen fluxes are expressed as a percentage of the dietary intake (10 mmol N·100 g BW^−1^·d^−1^). ^1^Total net intestinal absorption is composed of proximal absorption from the small intestine (f_absSI_−f_secSI_) and distal absorption from the colon and cecum (f_absCC_−f_UH_, see Figure 2 for model abbreviations). Nitrogen flux values were estimated from data in the literature and to comply with steady-states conditions for the whole system and each model compartment (see Supplementary Table 3 for more detail). In each tissue, the protein turnover flux corresponds to the protein synthesis flux, which is equal to the protein degradation flux (f_s_ = f_d_).
(TIF)

Figure S2 Comparison of tissue amino acid fluxes in the model calibrated under standard conditions. (TIF) Comparison of the different inward and outward fluxes of amino acids (AA) in some characteristic tissues (muscle, A; skin, B; liver, C; and small intestine mucosa, D). The different inward fluxes correspond to intra-tissue AA production by protein breakdown (f_d_) and to intra-tissue AA transport from the peripheral circulation (f_in_ in all tissues), intestinal absorption (f_absSI_ in intestine), hepatic portal transfer and entero-hepatic urea recycling (f_PV_ and f_absCC_ in liver, respectively). The different outward fluxes correspond to AA utilization for protein synthesis (f_s_) and oxidation (f_ox_), and to extra-tissue AA transport towards the peripheral circulation (f_out_) or portal vein and intestinal lumen (f_PV_ and f_secSI_ in intestine, respectively). For each tissue, the protein synthesis (f_s_) and breakdown (f_d_) fluxes are equal and the flux of AA oxidation (f_ox_) represents 29% of all AA metabolic utilization for protein synthesis and oxidation (i.e., %ox = f_ox_/(f_ox_+f_s_) = 29%, and f_ox_ = (%ox/(1−%ox))·f_s_ = 0.41·f_s_). The importance of AA exchange fluxes between plasma and tissue (f_in_, f_out_ and f_PV_, the fluxes of AA transfer from plasma to tissue, from tissue to plasma and from intestine to liver through the portal vein, respectively) varies depending on the tissues concerned.
(TIF)

Figure S3 Predicted nitrogen isotopic trajectories in tissue proteins after a change in splanchnic nitrogen fluxes. (TIF) Kinetic evolutions of ^15^N enrichment (Δ^15^N) in various tissue proteins were obtained by simulating an instantaneous increase, at time 0, in splanchnic oxidation fluxes (increase in k_oxL_ and k_oxSI_ by 45%) together with a compensating increase in urea recycling fluxes (increase in k_UH_ and k_absCC_ by 85% and 66% respectively) to maintain the initial elemental steady state. Variations in Δ^15^N are expressed as relative changes, i.e., the percentage of the variation accomplished relative to the total difference between final and initial steady state Δ^15^N values (Δ^15^N changes (t) = (Δ^15^N(t)−Δ^15^N_initial_)/(Δ^15^N_final_−Δ^15^N_initial_)).
(TIF)

Figure S4 Variations in nitrogen isotopic abundances in liver and small intestine in relation to combined variations in splanchnic oxidation, urea recycling and peripheral delivery. (TIF) Variations in the ^15^N enrichment (Δ^15^N) of hepatic and intestinal proteins in response to counterbalanced, homeostatic changes in relative splanchnic oxidation (%ox, in %) and in the efficiencies of urea recycling (R, in % of urea production) and peripheral delivery (P, in % of dietary nitrogen intake), which may result from qualitative and/or quantitative variations in the dietary protein intake (Simulation #3). %ox is defined as a proportion of splanchnic amino acid utilization for protein synthesis and oxidation (%ox = f_oxSpl_/(f_oxSpl_+f_sSpl_), with f_oxSpl_ = f_oxL_+f_oxSI_ and f_sSpl_ = f_sL_+f_sPl_+f_sSI_), R = f_UH_/f_UP_ and P = f_outL_ (see Figure 2). Variations in %ox were simulated through changes in the k_oxSI_ and k_oxL_ parameter values, while variations in R and P were respectively achieved through changes in the k_UH_ and k_outL_ parameter values. The blue line corresponds to simulations for which a 0 to 71% increase in the initial %ox (i.e., %ox increasing from 29% to 50%), is entirely offset by a 0% to 148% increase in the initial R (i.e., R increasing from 18% to 45%), with no change in P. The red line corresponds to simulations for which a 28% to 71% increase in the initial %ox is counterbalanced by a decrease in P ranging from 0 to 13% (i.e., P decreasing from 250% to 216%), with R being fixed at 29% (i.e., increased by 60% compared to its initial value). The shaded area between the red and blue lines corresponds to intermediate scenarios under which the increase in %ox is counterbalanced to varying degrees by an increase in R (ranging from 60% to 148%) and a decrease in P (ranging from 0 to 13%). Within this area, a similar Δ^15^N variation (corresponding to a horizontal line) can be obtained for different combinations of R and P variations. Variations in Δ^15^N are expressed as the difference between final and initial steady state Δ^15^N values.
(TIF)

Figure S5 Variations in fecal and urinary nitrogen isotopic abundances in relation to urea recycling efficiency. (TIF) Model predicted variations in the ^15^N enrichment (Δ^15^N) of urinary urea and fecal nitrogen losses resulted from model simulations generated by varying the amino acid oxidation flux values and compensating by changing the urea recycling efficiency (f_UH_/f_UP_) and peripheral delivery (f_outL_) values to different degrees (Simulation #3). Variations in Δ^15^N are expressed as the difference between the steady state Δ^15^N values after and before this flux variation. Solid lines correspond to linear regressions: Δ^15^N variations were significantly correlated to urea recycling efficiency for fecal losses (correlation coefficient R^2^ = 0.97; with a slope of −0.9) but not for urinary urea (correlation coefficient R^2^ = 0.01).
(TIF)

Table S1 Model differential equations describing the state variables (N and δ^15^N) in the liver and muscle compartments.
(PDF)

Table S2 Experimental data (N and Δ^15^N) used for model calibration.
(PDF)

Table S3 Data from the literature, hypothesis and equations used for model calibration and estimated values for model parameters.
(PDF)

Table S4 Isotope half-lives (t_50%_) and times to reach an isotopic equilibrium (t_95%_) in rat tissues following a dietary or metabolic change.
(PDF)

Table S5 Comparison of our experimental Δ^15^N values with those from the literature in various rodent tissues.
(PDF)

Text S1 Physiological plausibility of simulated values for fractionation factors ε.
(DOCX)

Text S2 Calculations of nitrogen (N) amounts in model compartments.
(DOCX)

Text S3 Definitions and relationships between the model state variables and parameters and details on the derivation of model equations.
(PDF)
