# Supplementary figures and images for: Natural Isotopic Signatures of Variations in Body Nitrogen Fluxes: A Compartmental Model Analysis
Source: PLoS Comput Biol. 2014 Oct 2;10(10):e1003865. doi: 10.1371/journal.pcbi.1003865 (PMC4183419; doi:10.1371/journal.pcbi.1003865)

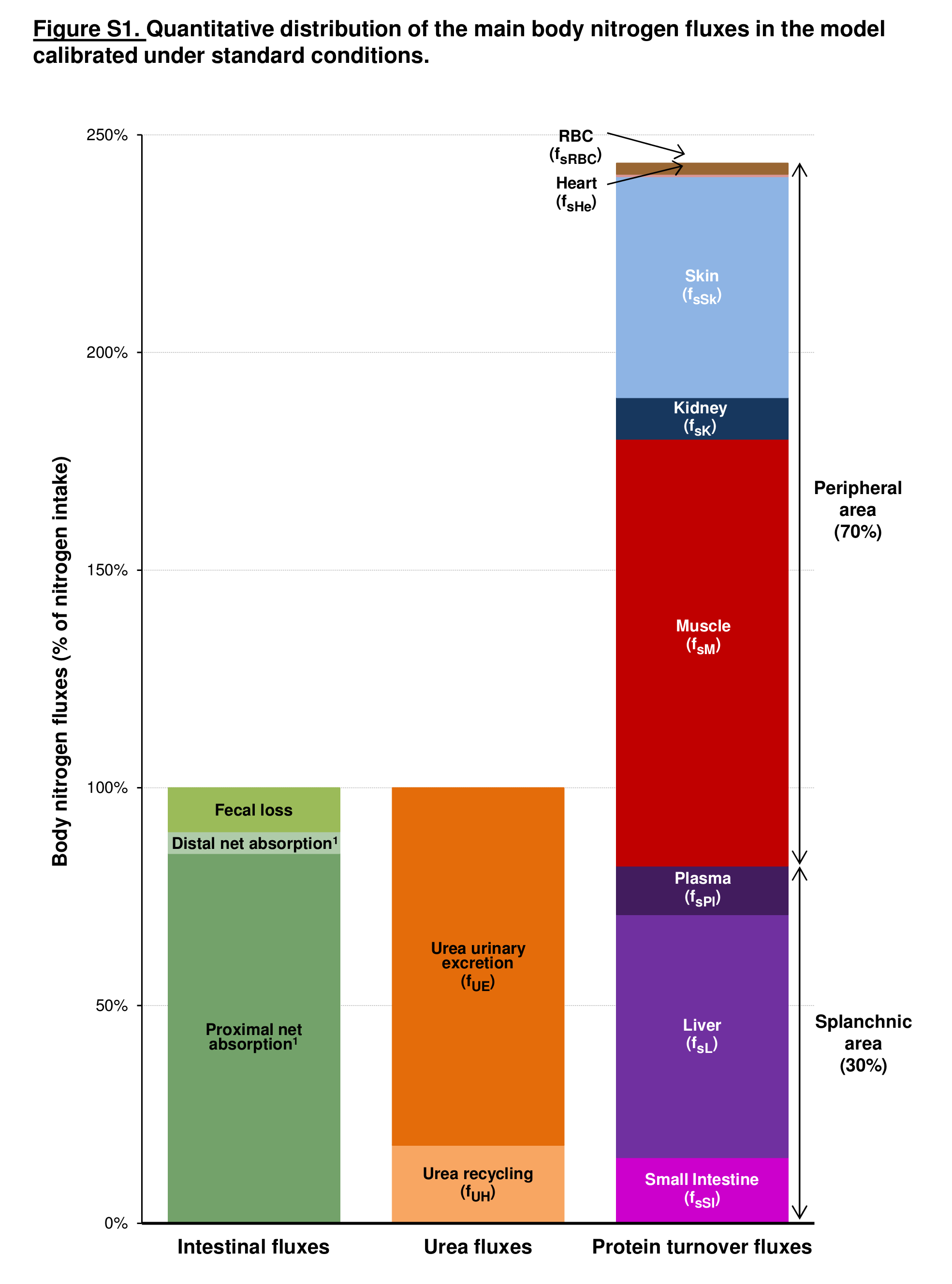

Supplement: Figure S1 — Quantitative distribution of the main body nitrogen fluxes in the model calibrated under standard conditions. (TIF) Nitrogen fluxes are expressed as a percentage of the dietary intake (10 mmol N·100 g BW−1·d−1). 1Total net intestinal absorption is composed of proximal absorption from the small intestine (fabsSI−fsecSI) and distal absorption from the colon and cecum (fabsCC−fUH, see Figure 2 for model abbreviations). Nitrogen flux values were estimated from data in the literature and to comply with steady-states conditions for the whole system and each model compartment (see Supplementary Table 3 for more detail). In each tissue, the protein turnover flux corresponds to the protein synthesis flux, which is equal to the protein degradation flux (fs = fd). (TIF) [file pcbi.1003865.s001.tif]

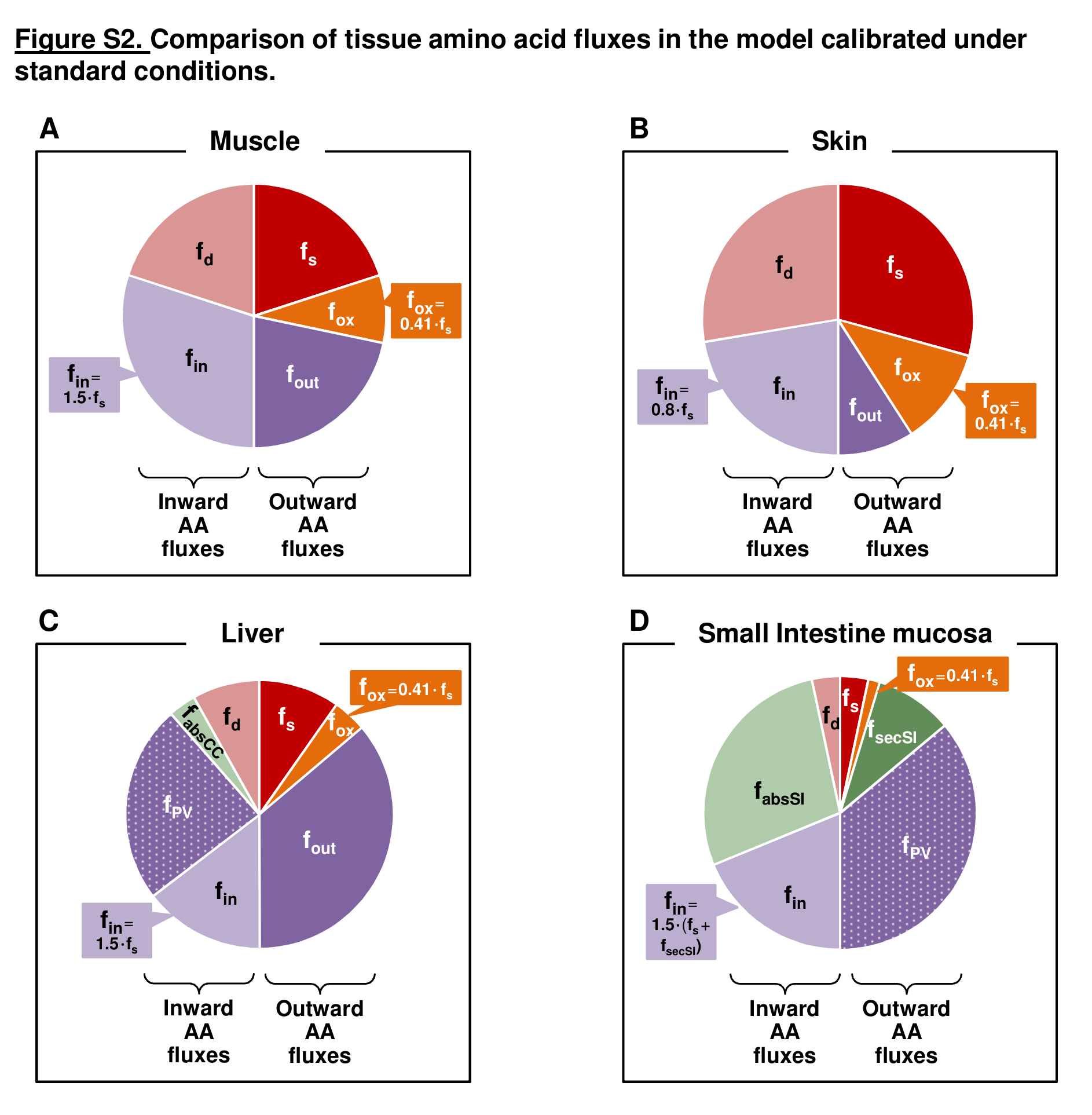

Supplement: Figure S2 — Comparison of tissue amino acid fluxes in the model calibrated under standard conditions. (TIF) Comparison of the different inward and outward fluxes of amino acids (AA) in some characteristic tissues (muscle, A; skin, B; liver, C; and small intestine mucosa, D). The different inward fluxes correspond to intra-tissue AA production by protein breakdown (fd) and to intra-tissue AA transport from the peripheral circulation (fin in all tissues), intestinal absorption (fabsSI in intestine), hepatic portal transfer and entero-hepatic urea recycling (fPV and fabsCC in liver, respectively). The different outward fluxes correspond to AA utilization for protein synthesis (fs) and oxidation (fox), and to extra-tissue AA transport towards the peripheral circulation (fout) or portal vein and intestinal lumen (fPV and fsecSI in intestine, respectively). For each tissue, the protein synthesis (fs) and breakdown (fd) fluxes are equal and the flux of AA oxidation (fox) represents 29% of all AA metabolic utilization for protein synthesis and oxidation (i.e., %ox = fox/(fox+fs) = 29%, and fox = (%ox/(1−%ox))·fs = 0.41·fs). The importance of AA exchange fluxes between plasma and tissue (fin, fout and fPV, the fluxes of AA transfer from plasma to tissue, from tissue to plasma and from intestine to liver through the portal vein, respectively) varies depending on the tissues concerned. (TIF) [file pcbi.1003865.s002.tif]

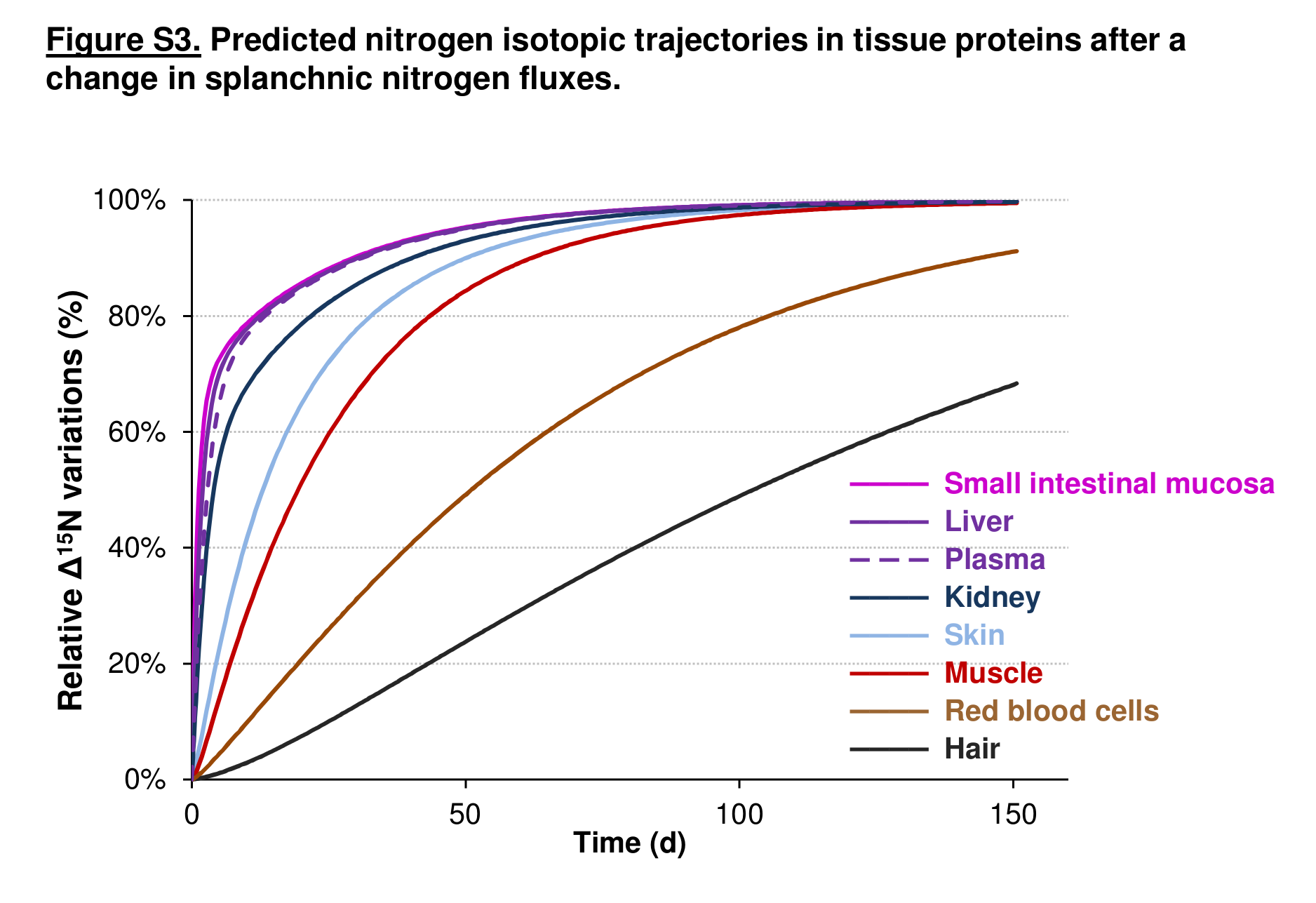

Supplement: Figure S3 — Predicted nitrogen isotopic trajectories in tissue proteins after a change in splanchnic nitrogen fluxes. (TIF) Kinetic evolutions of 15N enrichment (Δ15N) in various tissue proteins were obtained by simulating an instantaneous increase, at time 0, in splanchnic oxidation fluxes (increase in koxL and koxSI by 45%) together with a compensating increase in urea recycling fluxes (increase in kUH and kabsCC by 85% and 66% respectively) to maintain the initial elemental steady state. Variations in Δ15N are expressed as relative changes, i.e., the percentage of the variation accomplished relative to the total difference between final and initial steady state Δ15N values (Δ15N changes (t) = (Δ15N(t)−Δ15Ninitial)/(Δ15Nfinal−Δ15Ninitial)). (TIF) [file pcbi.1003865.s003.tif]

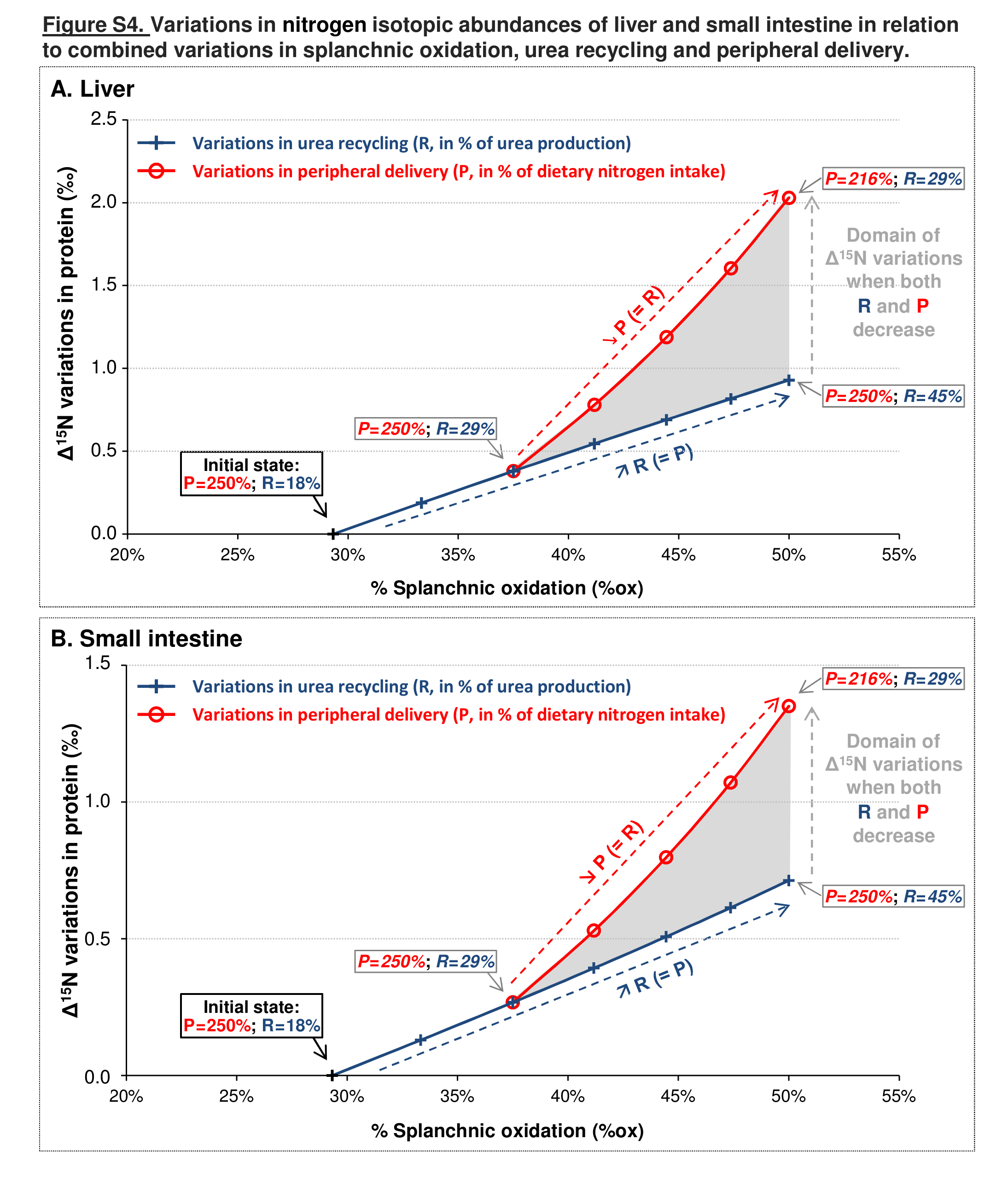

Supplement: Figure S4 — Variations in nitrogen isotopic abundances in liver and small intestine in relation to combined variations in splanchnic oxidation, urea recycling and peripheral delivery. (TIF) Variations in the 15N enrichment (Δ15N) of hepatic and intestinal proteins in response to counterbalanced, homeostatic changes in relative splanchnic oxidation (%ox, in %) and in the efficiencies of urea recycling (R, in % of urea production) and peripheral delivery (P, in % of dietary nitrogen intake), which may result from qualitative and/or quantitative variations in the dietary protein intake (Simulation #3). %ox is defined as a proportion of splanchnic amino acid utilization for protein synthesis and oxidation (%ox = foxSpl/(foxSpl+fsSpl), with foxSpl = foxL+foxSI and fsSpl = fsL+fsPl+fsSI), R = fUH/fUP and P = foutL (see Figure 2). Variations in %ox were simulated through changes in the koxSI and koxL parameter values, while variations in R and P were respectively achieved through changes in the kUH and koutL parameter values. The blue line corresponds to simulations for which a 0 to 71% increase in the initial %ox (i.e., %ox increasing from 29% to 50%), is entirely offset by a 0% to 148% increase in the initial R (i.e., R increasing from 18% to 45%), with no change in P. The red line corresponds to simulations for which a 28% to 71% increase in the initial %ox is counterbalanced by a decrease in P ranging from 0 to 13% (i.e., P decreasing from 250% to 216%), with R being fixed at 29% (i.e., increased by 60% compared to its initial value). The shaded area between the red and blue lines corresponds to intermediate scenarios under which the increase in %ox is counterbalanced to varying degrees by an increase in R (ranging from 60% to 148%) and a decrease in P (ranging from 0 to 13%). Within this area, a similar Δ15N variation (corresponding to a horizontal line) can be obtained for different combinations of R and P variations. Variations in Δ15N are expressed as the difference between fi [file pcbi.1003865.s004.tif]

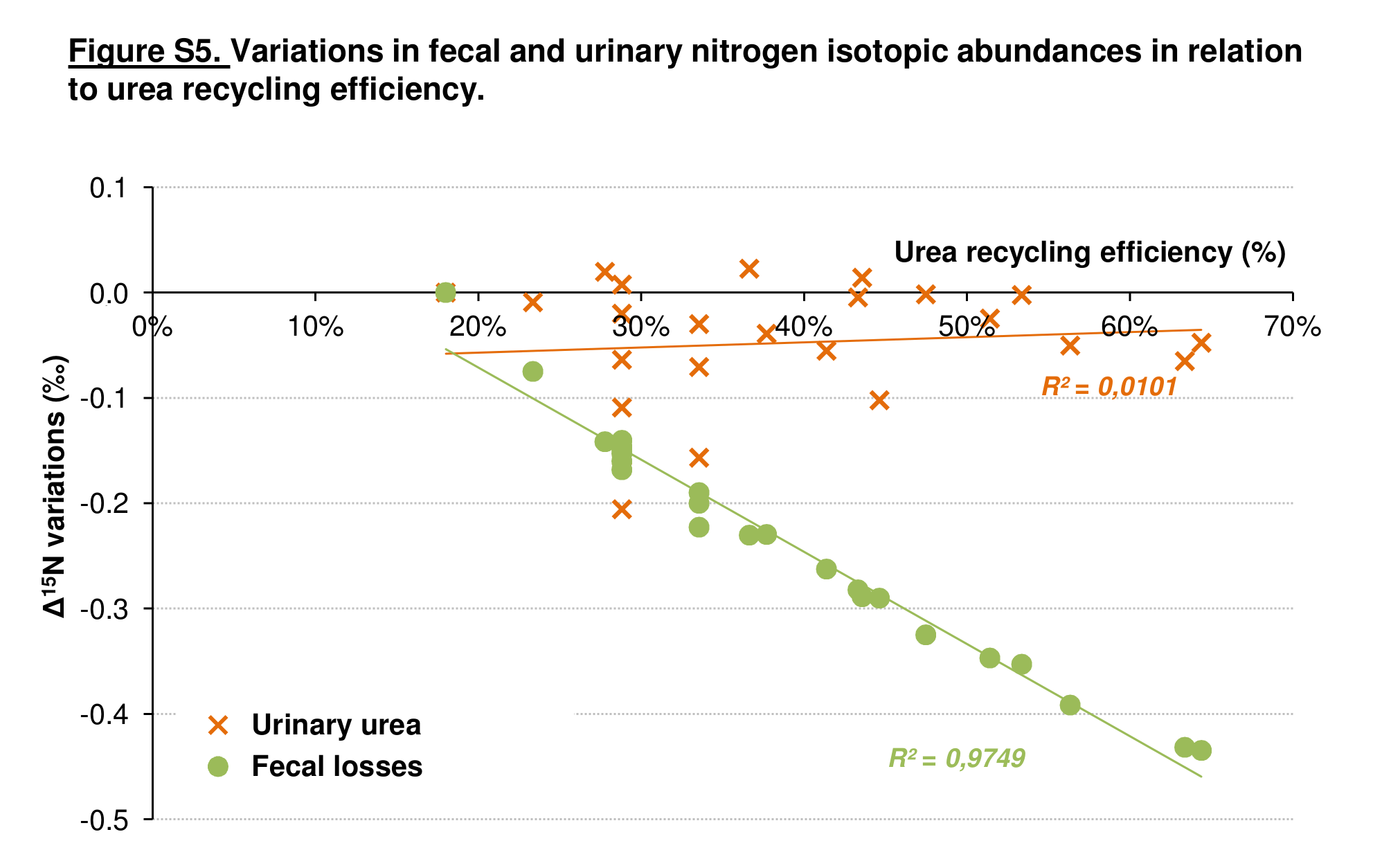

Supplement: Figure S5 — Variations in fecal and urinary nitrogen isotopic abundances in relation to urea recycling efficiency. (TIF) Model predicted variations in the 15N enrichment (Δ15N) of urinary urea and fecal nitrogen losses resulted from model simulations generated by varying the amino acid oxidation flux values and compensating by changing the urea recycling efficiency (fUH/fUP) and peripheral delivery (foutL) values to different degrees (Simulation #3). Variations in Δ15N are expressed as the difference between the steady state Δ15N values after and before this flux variation. Solid lines correspond to linear regressions: Δ15N variations were significantly correlated to urea recycling efficiency for fecal losses (correlation coefficient R2 = 0.97; with a slope of −0.9) but not for urinary urea (correlation coefficient R2 = 0.01). (TIF) [file pcbi.1003865.s005.tif]
