# Supplementary material for: Natural Isotopic Signatures of Variations in Body Nitrogen Fluxes: A Compartmental Model Analysis
Source: PLoS Comput Biol. 2014 Oct 2;10(10):e1003865. doi: 10.1371/journal.pcbi.1003865 (PMC4183419; doi:10.1371/journal.pcbi.1003865)
Supplement: Table S1 — Model differential equations describing the state variables (N and δ15N) in the liver and muscle compartments. (PDF) [file pcbi.1003865.s006.pdf]

Table S1. **Model differential equations describing the state variables (N and δ^{15}N) in the liver and muscle compartments.**

Compart- -ment	State variable	ODE	Particular values at the elemental steady state	Particular values at the elemental and isotopic steady states
Liver free amino acids (L_{AA})	N size (L_{AA})	$\frac{\mathbf{dL_{AA}(t)}}{\mathbf{dt}} = f_{PV}(t) + f_{absCC}(t) + f_{inL}(t) - f_{outL}(t) - f_{oxL}(t) + f_{dL}(t) - f_{sL}(t) - f_{sPl}(t)$	$f_{PV} + f_{absCC} + f_{inL} = f_{outL} + f_{oxL} + f_{sPl}$ and $\mathbf{L_{AA}} = \frac{k_{PV} \cdot SI_{AA} + k_{absCC} \cdot CC_L + k_{inL} \cdot AA_{Pl}}{k_{outL} + k_{oxL} + k_{sPl}}$	
	δ^{15}N (δL_{AA})	$\frac{\mathbf{d\delta L_{AA}(t)}}{\mathbf{dt}} = \frac{f_{PV}(t)}{L_{AA}(t)} \cdot (\delta SI_{AA}(t) - \delta L_{AA}(t) + \varepsilon_{PV}) + \frac{f_{absCC}(t)}{L_{AA}(t)} \cdot (\delta CC_L(t) - \delta L_{AA}(t) + \varepsilon_{absCC}) + \frac{f_{inL}(t)}{L_{AA}(t)} \cdot (\delta PL_{AA}(t) - \delta L_{AA}(t)) + \frac{f_{dL}(t)}{L_{AA}(t)} \cdot (\delta L_P(t) - \delta L_{AA}(t)) - \frac{f_{sL}(t)}{L_{AA}(t)} \cdot \varepsilon_{sL} - \frac{f_{sPl}(t)}{L_{AA}(t)} \cdot \varepsilon_{sPl} - \frac{f_{oxL}(t)}{L_{AA}(t)} \cdot \varepsilon_{oxL} - \frac{f_{outL}(t)}{L_{AA}(t)} \cdot \varepsilon_{outL}$	$\frac{\mathbf{d\delta L_{AA}(t)}}{\mathbf{dt}} = \frac{f_{PV}}{L_{AA}} \cdot (\delta SI_{AA}(t) - \delta L_{AA}(t) + \varepsilon_{PV}) + \frac{f_{absCC}}{L_{AA}} \cdot (\delta CC_L(t) - \delta L_{AA}(t) + \varepsilon_{absCC}) + \frac{f_{inL}}{L_{AA}} \cdot (\delta PL_{AA}(t) - \delta L_{AA}(t)) + \frac{f_{sL}}{L_{AA}} \cdot (\delta L_P(t) - \delta L_{AA}(t) - \varepsilon_{sL}) - \frac{f_{sPl}}{L_{AA}} \cdot \varepsilon_{sPl} - \frac{f_{oxL}}{L_{AA}} \cdot \varepsilon_{oxL} - \frac{f_{outL}}{L_{AA}} \cdot \varepsilon_{outL}$	$\mathbf{\delta L_{AA}} = \frac{f_{PV} \cdot (\delta SI_{AA} + \varepsilon_{PV}) + f_{absCC} \cdot (\delta CC_L + \varepsilon_{absCC}) + f_{inL} \cdot \delta PL_{AA} - f_{sPl} \cdot \varepsilon_{sPl} - f_{oxL} \cdot \varepsilon_{oxL} - f_{outL} \cdot \varepsilon_{outL}}{f_{sPl} + f_{oxL} + f_{outL}}$
Liver proteins (L_P)	N size (L_P)	$\frac{\mathbf{dL_P(t)}}{\mathbf{dt}} = f_{sL}(t) - f_{dL}(t)$	$f_{sL} = f_{dL}$ and $\mathbf{L_P} = \frac{ks_L}{kd_L} \cdot L_{AA}$	
	δ^{15}N (δL_P)	$\frac{\mathbf{d\delta L_P(t)}}{\mathbf{dt}} = \frac{f_{sL}(t)}{L_P(t)} \cdot (\delta L_{AA}(t) - \delta L_P(t) + \varepsilon_{sL})$	$\frac{\mathbf{d\delta L_P(t)}}{\mathbf{dt}} = \frac{f_{sL}}{L_P} \cdot (\delta L_{AA}(t) - \delta L_P(t) + \varepsilon_{sL})$	$\mathbf{\delta L_P} = \delta L_{AA} + \varepsilon_{sL}$ or $\mathbf{\delta L_P} = \frac{f_{PV} \cdot (\delta SI_{AA} + \varepsilon_{PV}) + f_{absCC} \cdot (\delta CC_L + \varepsilon_{absCC}) + f_{inL} \cdot \delta PL_{AA} - f_{sPl} \cdot \varepsilon_{sPl} - f_{oxL} \cdot \varepsilon_{oxL} - f_{outL} \cdot \varepsilon_{outL}}{f_{sPl} + f_{oxL} + f_{outL}} + \varepsilon_{sL}$

Ordinary differential equations (ODE) for the N and δ^{15}N state variables were obtained by applying the mass conservation principle to the total N amount and the ^{15}N amount respectively, in each compartment. Particular values at elemental steady state (in green) were obtained by considering that total N amounts in compartments were constant ($dN/dt = 0$) and particular values at isotopic steady state were obtained by considering that δ^{15}N values in compartments were constant ($(d\delta^{15}\ N)/dt = 0$).

Table S1. Model differential equations describing the state variables (N and $\delta^{15}N$) in the liver and muscle compartments.

Compart- -ment	State variable	ODE	Particular values at the elemental steady state	Particular values at the elemental and isotopic steady states
Muscle free amino acids (M_{AA})	N size (M_{AA})	$\frac{dM_{AA}(t)}{dt} = f_{inM}(t) - f_{outM}(t) - f_{oxM}(t) + f_{dM}(t) - f_{sM}(t)$		
			$f_{inM} = f_{outM} + f_{oxM}$ and $M_{AA} = \frac{kin_M}{kout_M + kox_M} \cdot Pl_{AA}$	
	$\delta^{15}N$ (δM_{AA})	$\frac{d\delta M_{AA}(t)}{dt} = \frac{f_{inM}(t)}{M_{AA}(t)} \cdot (\delta Pl_{AA}(t) - \delta M_{AA}(t)) + \frac{f_{dM}(t)}{M_{AA}(t)} \cdot (\delta M_P(t) - \delta M_{AA}(t)) - \frac{f_{oxM}(t)}{M_{AA}(t)} \cdot \varepsilon_{oxM} - \frac{f_{sM}(t)}{M_{AA}(t)} \cdot \varepsilon_{sM}$		
			$\frac{d\delta M_{AA}(t)}{dt} = \frac{f_{inM}}{M_{AA}} \cdot (\delta Pl_{AA}(t) - \delta M_{AA}(t)) + \frac{f_{sM}}{M_{AA}} \cdot (\delta M_P(t) - \delta M_{AA}(t) - \varepsilon_{sM}) - \frac{f_{oxM}}{M_{AA}} \cdot \varepsilon_{oxM}$	
				$\delta M_{AA} = \delta Pl_{AA} - \frac{f_{oxM}}{f_{inM}} \cdot \varepsilon_{oxM}$ or $\delta M_{AA} = \delta Pl_{AA} - \frac{f_{oxM}}{f_{oxM} + f_{outM}} \cdot \varepsilon_{oxM}$
Muscle proteins (M_P)	N size (M_P)	$\frac{dM_P(t)}{dt} = f_{sM}(t) - f_{dM}(t)$		
			$f_{sM} = f_{dM}$ and $M_P = \frac{ks_M}{kd_M} \cdot M_{AA}$	
	$\delta^{15}N$ (δM_P)	$\frac{d\delta M_P(t)}{dt} = \frac{f_{sM}(t)}{M_P(t)} \cdot (\delta M_{AA}(t) - \delta M_P(t) + \varepsilon_{sM})$		
			$\frac{d\delta M_P(t)}{dt} = \frac{f_{sM}}{M_P} \cdot (\delta M_{AA}(t) - \delta M_P(t) + \varepsilon_{sM})$	
				$\delta M_P = \delta M_{AA} + \varepsilon_{sM}$ or $\delta M_P = \delta Pl_{AA} - \frac{f_{oxM}}{f_{inM}} \cdot \varepsilon_{oxM} + \varepsilon_{sM}$

Ordinary differential equations (ODE) for the N and $\delta^{15}N$ state variables were obtained by applying the mass conservation principle to the total N amount and the ^{15}N amount respectively, in each compartment. Particular values at elemental steady state (in green) were obtained by considering that total N amounts in compartments were constant ($dN/dt = 0$) and particular values at isotopic steady state were obtained by considering that $\delta^{15}N$ values in compartments were constant ($(d\delta^{15} N)/dt = 0$).
