# Supplementary material for: Natural Isotopic Signatures of Variations in Body Nitrogen Fluxes: A Compartmental Model Analysis
Source: PLoS Comput Biol. 2014 Oct 2;10(10):e1003865. doi: 10.1371/journal.pcbi.1003865 (PMC4183419; doi:10.1371/journal.pcbi.1003865)
Supplement: Table S2 — Experimental data (N and Δ15N) used for model calibration. (PDF) [file pcbi.1003865.s007.pdf]

Table S2. Experimental data (N and $\Delta^{15}N$) used for model calibration.

Nitrogen body pool	Model compartment	Experimental data	
		**N** *(mmol·100g BW^{-1})*	**$\Delta^{15}N$** *(‰)*
Stomach lumen content	**StL**	0.2	≈ 0†
Small Intestine lumen content	**SIL**	0.2	+1.03
Caeco-colonic lumen content	**CCL**	0.3	+1.01
Small Intestinal mucosa			
AA fraction	**SI_{AA}**	0.060	+1.36
P fraction	**SI_{P}**	0.85	+2.59
Liver			
AA fraction	**L_{AA}**	0.22	+1.91
P fraction	**L_{P}**	5.2	+4.45
Muscle			
AA fraction	**M_{AA}**	5.90	-1.46
P fraction	**M_{P}**	88.9	+3.36
Skin			
AA fraction	**Sk_{AA}**	0.40	+1.11
P fraction	**Sk_{P}**	20.2	+2.70
Kidney			
AA fraction	**K_{AA}**	0.040	+2.41
P fraction	**K_{P}**	0.90	+3.05
Heart			
AA fraction	**He_{AA}**	0.022	+0.58
P fraction	**He_{P}**	0.35	+4.70
Red Blood Cell			
AA fraction	**RBC_{AA}**	0.072	+1.25†
P fraction	**RBC_{P}**	8.0	+2.68
Hair	**Ha**	38.9	+2.30
Plasma			
AA fraction	**Pl_{AA}**	0.015	+0.25
P fraction	**Pl_{P}**	1.85	+5.06
Body urea	**BU**	0.9	-1.05
Urinary urea	**UrU**	x	-0.10
Urinary NH_4^+	**UrNH4**	x	+0.87

N, total amount of nitrogen (^{14}N+^{15}N) and $\Delta^{15}N$, natural ^{15}N enrichment relative to the diet, were measured in various tissues and nitrogen fractions; AA, free amino acids; P, proteins; † values not measured experimentally but hypothesized; x, values not used for model calibration.
