# Supplementary material for: Natural Isotopic Signatures of Variations in Body Nitrogen Fluxes: A Compartmental Model Analysis
Source: PLoS Comput Biol. 2014 Oct 2;10(10):e1003865. doi: 10.1371/journal.pcbi.1003865 (PMC4183419; doi:10.1371/journal.pcbi.1003865)
Supplement: Table S3 — Data from the literature, hypothesis and equations used for model calibration and estimated values for model parameters. (PDF) [file pcbi.1003865.s008.pdf]

Table S3. Data from literature, hypothesis and equations used for model calibration and estimated values for model parameters.

	Model nitrogen fluxes			parameter k	parameter Ů
**notation**	estimated value (mmol N·100g BW·d^{-1})		*Hypothesis used or mass balance constraints*	estimated value (d^{-1})	estimated value (ă)
Intake and loss fluxes					
$\mathbf{N_{intake}}$	10	*(C)*[A]			Ø
$\mathbf{N_{losses}}$	10	*(SS$_{WB}$)*	$N_{intake} = N_{losses} = f_{UE} + f_{NH4} + f_{FL} + f_{lHa} + f_{lSk}$		
$\mathbf{f_{lHa}}$	0.25	*(C & SS$_{WB}$)*	$f_{lHa}+f_{lSk}$ = 3%[ÀÀ] N_{losses}	0.006	Ø
$\mathbf{f_{lSk}}$	0.05	*(C & SS$_{WB}$)*		0.002	Ø
$\mathbf{f_{FL}}$	1.0	*(C & SS$_{WB}$)*	10%[A] x ($f_{UE} + f_{NH4} + f_{FL}$) = 10%[A] x (97% N_{intake})	2.00	Ø
$\mathbf{f_{UE}}$	8.2	*(C & SS$_{WB}$)*	95%[A] x ($f_{UE} + f_{NH4}$) = 90%[A] x (97% N_{intake})	7.52	+1.45
$\mathbf{f_{NH4}}$	0.5	*(C & SS$_{WB}$)*	5%[A] x ($f_{UE} + f_{NH4}$) = 90%[A] x (97% N_{intake})	10.40	-0.49
Gastrointestinal fluxes					
$\mathbf{f_{tSt}}$	10	*(C)*[AA]		14.29	Ø
$\mathbf{f_{absSI}}$	12.7	*(C)*[AA]		31.75	Ø
$\mathbf{f_{secSI}}$	4.2	*(C)*[AA]		30.00	+0.83
$\mathbf{f_{tSI}}$	1.5	*(SS$_{SIL}$)*	$f_{tSI} = N_{intake} + f_{secSI} - f_{absSI}$	3.75	Ø
$\mathbf{f_{absCC}}$	2.3	*(SS$_{CCL}$)*	$f_{absCC} = N_{intake} + f_{secSI} + f_{UH} - f_{absSI} - f_{FL}$	4.60	-1.71
$\mathbf{f_{UH}}$	1.8	*(SS$_{BU}$)*	f_{UH} = ×$_T f_{oxT}$ ï f_{UE},<br>with ×$_T f_{oxT} = f_{UP}$ = 100%[À] x N_{intake}	1.70	Ø
Tissue protein synthesis and breakdown fluxes					
$\mathbf{f_{sSI}}$	1.52	*(C)*	$f_{sSI} = FSR_{SI} \times SI_P$, with FSR_{SI}=150%·d^{-1}[AA]	10.82	+1.68
$\mathbf{f_{sL}}$	5.58	*(C)*	$f_{sL} = FSR_L \times L_P$, with FSR_L=100%·d^{-1}[AA]	15.33	+3.12
$\mathbf{f_{sPl}}$	1.12	*(C)*	$f_{sPl} = FSR_{Pl} \times Pl_P$, with FSR_{Pl}=58%·d^{-1}[AA]	3.08	+3.76
$\mathbf{f_{sSk}}$	5.08	*(C)*	$f_{sSk} = FSR_{Sk} \times Sk_P$, with FSR_{Sk}=25%·d^{-1}[AA]	11.03	+1.88
$\mathbf{f_{sM}}$	9.80	*(C)*	$f_{sM} = FSR_M \times M_P$, with FSR_M=11%·d^{-1}[AA]	1.51	+4.77
$\mathbf{f_{sK}}$	0.96	*(C)*	$f_{sK} = FSR_K \times K_P$, with FSR_K=103%·d^{-1}[AA]	19.96	+0.99
$\mathbf{f_{sHe}}$	0.06	*(C)*	$f_{sHe} = FSR_{He} \times He_P$, with FSR_{He}=16%·d^{-1}[AA]	2.24	+4.31
$\mathbf{f_{sRBC}}$	0.24	*(C)*	$f_{sRBC} = FSR_{RBC} \times RBC_P$, with FSR_{RBC}=3%·d^{-1}[AA]	2.89	+2.71
$\mathbf{f_{sHa}}$	0.25	*(SS$_{Ha}$)*	$f_{sHa} = f_{lHa}$	0.54	+1.49
$\mathbf{f_{dSI}}$	1.52	*(SS$_{SIP}$)*	$f_{dSI} = f_{sSI}$	1.50	Ø
$\mathbf{f_{dL}}$	5.60	*(SS$_{LP}$)*	$f_{dL} = f_{sL}$	1.00	Ø
$\mathbf{f_{dPl}}$	1.12	*(SS$_{SIP}$)*	$f_{dPl} = f_{sPl}$	0.58	Ø
$\mathbf{f_{dSk}}$	5.03	*(SS$_{PlP}$)*	$f_{dSk} = f_{sSk}$ ï f_{lSk}	0.25	Ø
$\mathbf{f_{dM}}$	9.80	*(SS$_{MP}$)*	$f_{dM} = f_{sM}$	0.11	Ø
$\mathbf{f_{dK}}$	0.96	*(SS$_{KP}$)*	$f_{dK} = f_{sK}$	1.03	Ø
$\mathbf{f_{dHe}}$	0.06	*(SS$_{HeP}$)*	$f_{dHe} = f_{sHe}$	0.16	Ø
$\mathbf{f_{dRBC}}$	0.24	*(SS$_{RBCP}$)*	$f_{dRBC} = f_{sRBC}$	0.03	Ø

Model nitrogen fluxes				parameter k	parameter Ů
**notation**	estimated value (mmol N·100g BW·d^{-1})		*Hypothesis used or mass balance constraints*	estimated value (d^{-1})	estimated value (ă)
Tissue amino acid oxidation fluxes					
$\mathbf{f_{oxSI}}$	0.63	(C)	$f_{oxSI} = f_{sSI}$ x [%ox/(1-%ox)], with %ox = 29%[1]	4.49	-5.77
$\mathbf{f_{oxL}}$	2.78	(C)	$f_{oxL} = f_{sL}$ x [%ox/(1-%ox)], with %ox = 29%[1]	7.64	-5.77
$\mathbf{f_{oxSk}}$	2.11	(C)	$f_{oxSk} = f_{sSk}$ x [%ox/(1-%ox)], with %ox = 29%[1]	4.58	-1.82
$\mathbf{f_{oxM}}$	4.07	(C)	$f_{oxM} = f_{sM}$ x [%ox/(1-%ox)], with %ox = 29%[1]	0.63	+5.08
$\mathbf{f_{oxK}}$	0.40	(C)	$f_{oxK} = f_{sK}$ x [%ox/(1-%ox)], with %ox = 29%[1]	8.28	-6.66
$\mathbf{f_{oxHe}}$	0.02	(C)	$f_{oxHe} = f_{sHe}$ x [%ox/(1-%ox)], with %ox = 29%[1]	0.93	-1.38
Amino acid exchange fluxes between plasma and tissue					
$\mathbf{f_{inSI}}$	8.57	(C)	$f_{inSI} = f_{sSI}$ x TI_{SI}, with TI_{SI} = 1,5[ÀÀ]	408.21	Ø
$\mathbf{f_{inL}}$	10.05	(C)	$f_{inL} = f_{sL}$ x TI_{L}, with TI_{L} = 1,5[ÀÀ]	478.53	Ø
$\mathbf{f_{inSk}}$	4.06	(C)	$f_{inSK} = f_{sSk}$ x TI_{Sk}, with TI_{Sk} = 0,8[ÀÀ]	193.33	Ø
$\mathbf{f_{inM}}$	14.70	(C)	$f_{inM} = f_{sM}$ x TI_{M}, with TI_{M} = 1,5[ÀÀ]	700.07	Ø
$\mathbf{f_{inK}}$	1.44	(C)	$f_{inK} = f_{sK}$ x TI_{K}, with TI_{K} = 1,5[ÀÀ]	68.42	Ø
$\mathbf{f_{inHe}}$	0.08	(C)	$f_{inHe} = f_{sHe}$ x TI_{He}, with TI_{He} = 1,5[ÀÀ]	4.00	Ø
$\mathbf{f_{inRBC}}$	0.36	(C)	$f_{inRBC} = f_{sRBC}$ x TI_{RBC}, with TI_{RBC} = 1,5[ÀÀ]	17.14	Ø
$\mathbf{f_{outL}}$	24.89	(SS_{LAA})	$f_{outL} = f_{inL} - f_{oxL}$	68.39	Ø
$\mathbf{f_{outSk}}$	1.66	(SS_{SkAA})	$f_{outSk} = f_{inSk} - f_{oxSk}$	3.60	Ø
$\mathbf{f_{outM}}$	10.64	(SS_{MAA})	$f_{outM} = f_{inM}$ ï f_{oxM}	1.64	Ø
$\mathbf{f_{outK}}$	0.54	(SS_{KAA})	$f_{outK} = f_{inK}$ ï f_{oxK}	11.24	Ø
$\mathbf{f_{outHe}}$	0.06	(SS_{HeAA})	$f_{outHe} = f_{inHe}$ ï f_{oxHe}	2.43	Ø
$\mathbf{f_{outRBC}}$	0.36	(SS_{RBCAA})	$f_{outRBC} = f_{inRBC}$	4.34	Ø
$\mathbf{f_{PV}}$	16.44	(SS_{SIAA})	$f_{PV} = f_{absSI} + f_{inSI}$ ï f_{secSI} ï f_{oxSI}	117.46	Ø

See Figure 2 and Table S2 for the model flux and compartment names, respectively. For each flux, letters in parentheses indicate how the flux value was determined under standard conditions: C, fluxes whose values were fixed based on experimental or literature data and SS_X, fluxes whose values were estimated in order to ensure the elementary steady state for compartment X or the whole body (WB); [À] values obtained from experimental data; [ÀÀ] values obtained from data in the literature; Ø, isotope effect assumed to be null; [1] %ox was supposed to be the same for all tissues and was calculated so that f_{UP} = ×$_T$ f_{oxT} = 100% N_{intake}, that is to say %ox = f_{UP} / (×$_T$ (f_{sT}) + f_{UP}); %ox, proportion of tissue amino acids that are oxidized rather than used for protein synthesis (%ox = f_{ox} / (f_s + f_{ox})); f_{UP}, total body urea production resulting from tissue amino acid catabolism.
