# Supplementary material for: Natural Isotopic Signatures of Variations in Body Nitrogen Fluxes: A Compartmental Model Analysis
Source: PLoS Comput Biol. 2014 Oct 2;10(10):e1003865. doi: 10.1371/journal.pcbi.1003865 (PMC4183419; doi:10.1371/journal.pcbi.1003865)
Supplement: Table S4 — Isotope half-lives (t50%) and times to reach an isotopic equilibrium (t95%) in rat tissues following a dietary or metabolic change. (PDF) [file pcbi.1003865.s009.pdf]

Table S4. Isotope half-live ($t_{50\%}$) and time to reach isotope equilibrium ($t_{95\%}$) in tissues following a dietary or metabolic change.

	Model predictions[1]				Data from literature after a dietary change[2]	
	after a dietary change		after a metabolic change			
Tissue	$t_{50\%}$	$t_{95\%}$	$t_{50\%}$	$t_{95\%}$	$t_{50\%}$	$t_{95\%}$
Small Intestine	1	37	1	49		
Liver	2	47	2	52	7 – 15	30 – 65
Kidney	4	57	4	61	8 – 22	31 – 96
Plasma	3	47	3	52	8	35 – 36
Muscle	19	79	20	83	16 – 46	68 – 200
RBC	51	179	52	181	29 – 34	127 – 147
Fur / Hair	122	486	103	265	65 – 70	281 – 303
Skin	13	67	13	71		

[1] $\Delta^{15}N$ evolutions in different tissue proteins were simulated after a dietary change (i.e., a change in diet $\delta^{15}N$) or a metabolic change (i.e., a change in the partitioning of splanchnic nitrogen fluxes such as described in additional file 2: Figure S3). The nitrogen isotope half-lives ($t_{50\%}$) and times to reach isotope equilibrium ($t_{95\%}$) were calculated from these simulated evolutions as the times needed to reach 50% and 95% of the final isotope equilibrium value, respectively, under each simulation. [2]data from the literature come from diet-shift experiments performed in rodents [1-4].
