# Supplementary material for: Natural Isotopic Signatures of Variations in Body Nitrogen Fluxes: A Compartmental Model Analysis
Source: PLoS Comput Biol. 2014 Oct 2;10(10):e1003865. doi: 10.1371/journal.pcbi.1003865 (PMC4183419; doi:10.1371/journal.pcbi.1003865)
Supplement: Table S5 — Comparison of our experimental Δ15N values with those from the literature in various rodent tissues. (PDF) [file pcbi.1003865.s010.pdf]

Table S5. Comparison of our experimental ȹ^{15}N values with those from the literature in various rodent tissues.

Tissue	Experimental ȹ^{15}N data	Data from literature[1]	
		ȹ^{15}N	References
**Small Intestine**	2.5	[1.4 ; 2.3]	[5, 6]
**Liver**	4.4	[1.1 ; 5.5]	[1, 2, 4, 6-11]
**Plasma**	5.0	[3.2 ; 4.9]	[2, 5, 11]
**Kidney**	3.0	[0.8 ; 2.8]	[2, 6, 8, 11]
**Muscle**	3.1	[1.1 ; 4.6]	[1, 2, 4, 7-12]
**Heart**	4.5	[2.7 ; 5.8]	[8, 11]
**RBC**	2.7	[1.2 ; 2.9]	[2, 11]
**Hair**	2.3	[-1.5 ; 4.1]	[2, 7, 12]
**Urine**	-0.1	[-2,9 ; -0,9]	[5, 13]
**Feces**	1	[1,4 ; 2,3]	[5, 13]

ȹ^{15}N values are calculated as the difference between the ŭ^{15}N of whole tissue and that of the diet (ȹ^{15}N = ŭ^{15}N$_{tissue}$ ï ŭ^{15}N$_{diet}$). For our experimental data, whole tissue ŭ^{15}N were calculated as the weighted average of the isotopic compositions of the free amino acid (AA) and protein (P) fractions (ŭ^{15}N = (n_{AA}·ŭ^{15}N$_{AA}$ + n_P·ŭ^{15}N$_P$) / (n_{AA} + n_P), where n_{AA} and n_P are respectively the amounts of nitrogen in the AA and P fractions). [1]Data from the literature come from studies performed in rodents.
