# Supplementary material for: Natural Isotopic Signatures of Variations in Body Nitrogen Fluxes: A Compartmental Model Analysis
Source: PLoS Comput Biol. 2014 Oct 2;10(10):e1003865. doi: 10.1371/journal.pcbi.1003865 (PMC4183419; doi:10.1371/journal.pcbi.1003865)
Supplement: Text S1 — Physiological plausibility of simulated values for fractionation factors ε. (DOCX) [file pcbi.1003865.s011.docx]

**Text S1. Physiological plausibility and interpretation of simulated values for the fractionation factors** **ε.**

- Fractionation factors associated with urea production and urinary excretion:

Although, based on experimental measurements, the δ^15^N of urinary urea was not significantly lower than that of the diet, body urea was ^15^N-deprived relative to urinary urea and to most of tissue amino acid (AA) compartments, except for muscle. In the calibrated model, these δ^15^N differences were respectively accounted for by a positive ε factor associated with urinary urea excretion (ε_UE_) and non-null fractionation factors associated with AA oxidation fluxes (ε_ox_), with negative values for all tissues except muscle. The experimentally observed differences in δ^15^N between tissue free AA and body urea, and the generally negative predicted ε_ox_ values, seem to agree with the common hypothesis in the literature that an isotopic fractionation is associated with deamination (reference [60] in the main text, [1]) leading to the formation of ^15^N-deprived urea and an accumulation of ^15^N-enriched AA in tissues (references [9, 11, 16, 17] in the main text). The ε_ox_ factors may reflect isotope effects that are indeed directly associated with the AA deamination processes, but also indirectly associated with the global intracellular metabolism of AA (transamination and interconversion of AA; reference [9] in the main text). However, these isotope effects did not explain the δ^15^N discrepancy between urinary urea and body urea, the fitting of which required a positive ε_UE_, which suggests the occurrence of additional fractionating steps during the urinary excretion process. In this respect, ε_UE_ may reflect an isotope effect associated with urea reabsorption in renal tubules. Therefore, as we understand and represent the system, the low δ^15^N value of the body urea is not only due to isotopic fractionation associated with AA deamination during urea synthesis, but is also probably dependent on the degree and efficiency of both renal tubular urea reabsorption and urea recycling.

The variations in estimated ε_ox_ values between tissues suggest that the nature of AA directed toward urea production through deamination and/or the intensity of intracellular AA transaminations and metabolic transformations vary as a function of the tissue concerned. This interpretation seems consistent with the fact that each tissue has specific metabolic requirements. Unlike all other sampled tissues, we surprisingly found a positive isotopic fractionation factor associated with the AA oxidation in muscle, which fitted with the observation of ^15^N-deprived free AA in muscle compared to plasma AA and body urea. This positive ε_ox_ factor may reflect the specifically large muscle release of glutamine and alanine, which are largely used as precursors for urea synthesis and are among the most ^15^N-enriched AA (reference [34] in the main text, [2, 3]). The presence of large amounts of creatine may also contribute to the very specific and low δ^15^N values observed in the muscle non-protein fraction.

- Fractionation factors associated with protein turnover in tissues:

The systematic ^15^N-enrichment of P *vs* AA fractions observed in each tissue was reproduced in our model by a positive isotope effect associated with P synthesis pathways. These positive ε_s_ factors identified for all tissues actually represent the net isotope effect associated with the protein synthesis and degradation processes and may therefore reflect either a selective utilization of ^15^N-enriched AA for protein synthesis, a preferential release of ^15^N-deprived AA from P degradation, or a combination of both. A few *in vitro* studies have consistently reported isotopic fractionation during peptide bond formation (reference [63] in the main text, [4, 5]) and hydrolysis [6, 7]. Positive ε_s_ factors may also indirectly reflect a preferential utilization of ^15^N-deprived AA in other intracellular metabolic pathways such as transaminations, leading to use of the remaining ^15^N-enriched AA for protein synthesis.

- Fractionation factors associated with intestinal absorption:

The model identified a negative fractionation factor (i.e., a preferential transfer of ^14^N) for the caeco-colonic absorption (ε_absCC_), which probably aggregates the isotope effects associated with urea hydrolysis by intestinal bacteria and the reabsorption of bacterial nitrogen compounds, which probably have lower δ^15^N values than endogenous nitrogen compounds. The model also identified a positive fractionation factor associated with the flux of endogenous secretions from intestinal AA towards the intestinal lumen (ε_secSI_), which agrees with the fact that these secretions mostly comprise gastrointestinal and pancreatic proteins which have a higher δ^15^N than intestinal AA. This positive ε_secSI_ may also reflect an opposite isotope effect associated with the reverse reaction, i.e. the preferential digestion and absorption of ^14^N, as suggested in some studies [8].

***References***

1. Werner RA, Schmidt HL (2002) The in vivo nitrogen isotope discrimination among organic plant compounds. Phytochemistry 61: 465-484.

2. Metges CC, Petzke KJ (1997) Measurement of 15N/14N isotopic composition in individual plasma free amino acids of human adults at natural abundance by gas chromatography-combustion isotope ratio mass spectrometry. Anal Biochem 247: 158-164.

3. Petzke KJ, Lemke S (2009) Hair protein and amino acid 13C and 15N abundances take more than 4 weeks to clearly prove influences of animal protein intake in young women with a habitual daily protein consumption of more than 1 g per kg body weight. Rapid Commun Mass Spectrom 23: 2411-2420.

4. Hiller DA, Singh V, Zhong M, Strobel SA (2011) A two-step chemical mechanism for ribosome-catalysed peptide bond formation. Nature 476: 236-239.

5. Seila AC, Okuda K, Nunez S, Seila AF, Strobel SA (2005) Kinetic isotope effect analysis of the ribosomal peptidyl transferase reaction. Biochemistry 44: 4018-4027.

6. Bada JL, Schoeninger MJ, Schimmelmann A (1989) Isotopic fractionation during peptide-bond hydroysis. Geochim Cosmochim Acta 53: 3337-3341.

7. Silfer JA, Engel MH, Macko SA (1992) Kinetic fractionation of stable carbon and nitrogen isotopes during peptide-bond hydrolysis - experimental-evidence and geochemical implications. Chem Geol 101: 211-221.

8. Hwang YT, Millar JS, Longstaffe FJ (2007) Do delta ^15^N and delta ^13^C values of feces reflect the isotopic composition of diets in small mammals? Can J Zool 85: 388-396.
