# Supplementary material for: Natural Isotopic Signatures of Variations in Body Nitrogen Fluxes: A Compartmental Model Analysis
Source: PLoS Comput Biol. 2014 Oct 2;10(10):e1003865. doi: 10.1371/journal.pcbi.1003865 (PMC4183419; doi:10.1371/journal.pcbi.1003865)
Supplement: Text S2 — Calculations of nitrogen (N) amounts in model compartments. (DOCX) [file pcbi.1003865.s012.docx]

# Text S2. Calculations of nitrogen (N) amounts in model compartments

The total N content in each sampled tissue fraction was calculated using the following equation:

N (mmol) = 1000 × (m × %DM/100 × %N/100) / 14,

where %N and %DM (%) are respectively the N and dry matter percentages measured in the sample, and m (g) the total mass of the tissue. The N percentage in each fraction of the tissue samples was determined using an elemental analyzer (EA 3000, Eurovector, Italy). The tissue dry matter percentage was estimated by weighing the tissue samples before and after freeze-drying.

To determine the total N amount in each compartment of the model, the following approximations were used:

- The total muscle mass was approximated as 95% of the “stripped” carcass mass [1];
- The N content in plasma P and AA fractions was calculated as the product of the N concentration in the fraction and the plasma volume (ml), which was estimated as: 0.0291 × BM + 2.54, where BM is the body mass (g) [2];
- The N amount in the body urea compartment (BU) was calculated from the plasma urea concentration (C_urea_, mmol·L^-1^ of urea-N), its volume of distribution (i.e., total body water, estimated as 62.3% of BM) [3], and a correction factor of 92% which represents the water content of the plasma [4], as follows:

BU (mmol N) = C_urea_ x 0.623 x BM / 0.92.

All N amounts in the model were expressed relative to body mass (mmol N·100g BM^-1^).

Based on 24-h dietary intake measurements, we estimated that the average daily intake was about 10 mmol N·100g BM^-1^.

In order to achieve a null daily N balance, we assumed that the sum of all cumulated N losses over 24-h was equal to the daily N intake. N losses were distributed as follows on the basis of data in the literature:

- - - - Miscellaneous N losses (hair and skin) were estimated at 3% of total N losses [5], with 20% from desquamation (f_lSk_ = 0.05 mmol N·100g BM^-1^·d^-1^ and f_lHa_ = 0.25 mmol N·100g BM^-1^·d^-1^, see Figure 2 for model flux abbreviations);
      - Based on previous measurements of N losses in rats of the same age and under similar conditions, N fecal losses were assumed to account for 10% of all urinary and fecal N losses (f_FL_ = 1 mmol N·100g BM^-1^·d^-1^);
      - The proportion of urea and ammonia in urinary losses was estimated from the relative urea and ammonia concentrations measured in urine samples: urea-N and ammonia-N were assumed to represent respectively 95% and 5% of urinary N losses: f_UE_ = 8.2 mmol N·100g BM^-1^·d^-1^ and f_NH4_ = 0.5 mmol N·100g BM^-1^·d^-1^.

***References:***

1. Lacroix M, Gaudichon C, Martin A, Morens C, Mathe V, Tome D, Huneau JF: **A long-term high-protein diet markedly reduces adipose tissue without major side effects in Wistar male rats.** *Am J Physiol Regul Integr Comp Physiol* 2004, **287:**R934-R942.

2. Bijsterbosch MK, Duursma AM, Bouma JMW, Gruber M: **The plasma-volume of the Wistar rat in relation to the body-weight.** *Experientia* 1981, **37:**381-382.

3. Boutry C, Fouillet H, Mariotti F, Blachier F, Tome D, Bos C: **Rapeseed and milk protein exhibit a similar overall nutritional value but marked difference in postprandial regional nitrogen utilization in rats.** *Nutr Metab (Lond)* 2011, **8:**52.

4. Sharp PE, LaRegina MC: **The laboratory rat.** In *The Laboratory Animal Pocket Reference Series.* CRC Press LLC; 1998

5. Rand WM, Pellett PL, Young VR: **Meta-analysis of nitrogen balance studies for estimating protein requirements in healthy adults.** *Am J Clin Nutr* 2003, **77:**109-127.
