# Supplementary material for: Natural Isotopic Signatures of Variations in Body Nitrogen Fluxes: A Compartmental Model Analysis
Source: PLoS Comput Biol. 2014 Oct 2;10(10):e1003865. doi: 10.1371/journal.pcbi.1003865 (PMC4183419; doi:10.1371/journal.pcbi.1003865)
Supplement: Text S3 — Definitions and relationships between the model state variables and parameters and details on the derivation of model equations. (PDF) [file pcbi.1003865.s013.pdf]

Text S3. Definitions and relationships between the model state variables and parameters and details on the derivation of model equations.

Each compartment i in the model is defined by its total nitrogen (N) content ($N_i = {}^{14}N_i + {}^{15}N_i$) and its isotopic enrichment ($\delta^{15}N_i$), which constitute the two state variables or species of the model. The amount of ^{15}N in compartment i can be calculated using the usual definition of $\delta^{15}N_i$:

$$\boldsymbol{\delta^{15}N_i} = \left(\frac{{}^{15}N_i/{}^{14}N_i}{R_{standard}} - 1\right) \times 1000$$

and by approximating the isotope ratio $^{15}N_i/^{14}N_i$ using the $^{15}N_i/N_i$ ratio (i.e., the fractional abundance), so that:

$$\boldsymbol{^{15}N_i} \approx \boldsymbol{N_i} \times \left(\frac{\boldsymbol{\delta N_i}}{1000} + 1\right) \times R_{std} \qquad \text{(E1)}$$

where R_{std} is the N isotope ratio for the internationally defined standard (atmospheric N_2, R_{std}=0.0036765).

In the model, for each compartment i, we defined inward and outward fluxes of total N ($f_{i,j}$ and $f_{j,i}$, respectively) and of the heavy N isotope ($^{15}f_{i,j}$ and $^{15}f_{j,i}$, respectively). These fluxes were assumed to follow mass action laws and were modeled according to the following equations:

$\mathbf{f_{i,j}}(t) = \mathbf{k_{i,j}} \times \mathbf{N_j}(t)$ and $\mathbf{^{15}f_{i,j}(t)} = \mathbf{^{15}k_{i,j}} \times \mathbf{^{15}N_j}(t)$for the inward flux from compartment j to i,

$\mathbf{f_{j,i}}(t) = \mathbf{k_{j,i}} \times \mathbf{N_i}(t)$ and $\mathbf{^{15}f_{j,i}(t)} = \mathbf{^{15}k_{j,i}} \times \mathbf{^{15}N_i}(t)$ for the outward flux from compartment i to j,

where $\mathbf{k_{j,i}}$ and $\mathbf{k_{i,j}}$ are the reaction rate constants (d^{-1}) for the corresponding N fluxes.

and $\mathbf{^{15}k_{j,i}}$ and $\mathbf{^{15}k_{i,j}}$ are the reaction rate constants (d^{-1}) for the corresponding ^{15}N fluxes.

We defined the fractionation factor $\varepsilon_{j,i}$, representing the isotopic effect associated with the flux $f_{j,i}$, as $\varepsilon_{j,i} = (\alpha_{j,i} - 1) \times 1000$, with $\alpha_{j,i} = {}^{15}k_{j,i}/k_{j,i}$. (E2)

Each compartment i is characterized by two differential equations describing respectively the kinetic evolution of its total N content (N_i) and its isotopic enrichment ($\delta^{15}N_i$).

Evolution of the total N content of compartment i:

According to the mass conservation principle, the evolution of total N content in compartment i is described by the following equation:

$$\frac{\mathbf{dN_i}(t)}{\mathbf{dt}} = \sum_{\mathbf{j}} \mathbf{f_{i,j}} - \sum_{\mathbf{j}} \mathbf{f_{j,i}} = \sum_{\mathbf{j}} \left(\mathbf{k_{i,j}} \times \mathbf{N_j}(t)\right) - \sum_{\mathbf{j}} \left(\mathbf{k_{j,i}} \times \mathbf{N_i}(t)\right) \qquad \text{(E3)}$$

Under the assumption of elemental steady state, the total N amounts in compartments are constants (i.e., $dN_i(t)/dt = 0$) and consequently Eq. E3 simplifies to:

$$\sum_j \mathbf{f}_{i,j} = \sum_j \mathbf{f}_{j,i}$$

Evolution of the isotopic enrichment of compartment i:

The equation describing the isotopic enrichment of compartment i was derived by applying the mass conservation principle to the heavy N isotope (^{15}N), as:

$$\frac{\mathbf{d^{15}N_i}(t)}{\mathbf{dt}} = \sum_j {}^{15}\mathbf{f}_{i,j}(t) - \sum_j {}^{15}\mathbf{f}_{j,i}(t) \qquad \text{(E4)}$$

Consequently, the evolution of the ^{15}N amount in compartment i can be expressed as:

$$\frac{\mathbf{d^{15}N_i}(t)}{\mathbf{dt}} = \sum_j \left({}^{15}\mathbf{k}_{i,j} \times {}^{15}\mathbf{N_j}(t)\right) - \sum_j \left({}^{15}\mathbf{k}_{j,i} \times {}^{15}\mathbf{N_i}(t)\right) \qquad \text{(E5)}$$

Using Eq. (E1) and applying the Leibniz rule to calculate the derivatives of (N_i x $\delta^{15}N_i$), Eq. E5 can be rewritten as:

$$\frac{N_i}{1000} \times \frac{\mathbf{d\delta^{15}N_i}(t)}{\mathbf{dt}} + \left(\frac{\boldsymbol{\delta^{15}N_i}}{1000} + 1\right) \times \frac{\mathbf{dN_i}(t)}{\mathbf{dt}} = \sum_j \left[{}^{15}\mathbf{k}_{i,j} \times \mathbf{N_j}(t) \times \left(\frac{\boldsymbol{\delta^{15}N_j}}{1000} + 1\right)\right] - \sum_j \left[{}^{15}\mathbf{k}_{j,i} \times \mathbf{N_i}(t) \times \left(\frac{\boldsymbol{\delta^{15}N_i}}{1000} + 1\right)\right]$$

By introducing the isotopic fractionation factors $\boldsymbol{\alpha}_{j,i}$ and $\boldsymbol{\alpha}_{i,j}$, defined according to Eq E2, the previous equation can be rewritten as:

$$\frac{N_i}{1000} \times \frac{\mathbf{d\delta^{15}N_i}(t)}{\mathbf{dt}} + \left(\frac{\boldsymbol{\delta^{15}N_i}}{1000} + 1\right) \times \frac{\mathbf{dN_i}(t)}{\mathbf{dt}} = \sum_j \left[\boldsymbol{\alpha}_{i,j} \times \mathbf{k}_{i,j} \times \mathbf{N_j}(t) \times \left(\frac{\boldsymbol{\delta^{15}N_j}}{1000} + 1\right)\right] - \sum_j \left[\boldsymbol{\alpha}_{j,i} \times \mathbf{k}_{j,i} \times \mathbf{N_i}(t) \times \left(\frac{\boldsymbol{\delta^{15}N_i}}{1000} + 1\right)\right]$$

which combined with Eq. E3, and using the mass action relations for the $f_{j,i}$ and $f_{i,j}$ fluxes, yields the following equation:

$$\frac{N_i}{1000} \times \frac{\mathbf{d\delta^{15}N_i}(t)}{\mathbf{dt}} = \sum_j \left[\mathbf{f}_{i,j}(t) \times \left(\boldsymbol{\alpha}_{i,j} - 1 + \boldsymbol{\alpha}_{i,j} \times \frac{\boldsymbol{\delta^{15}N_j}(t)}{1000} - \frac{\boldsymbol{\delta^{15}N_i}(t)}{1000}\right)\right] - \sum_j \left[\mathbf{f}_{j,i}(t) \times (\boldsymbol{\alpha}_{j,i} - 1) \times \left(\frac{\boldsymbol{\delta^{15}N_i}}{1000} + 1\right)\right]$$

Using the ε notation ($\alpha = \varepsilon/1000 + 1$) and assuming that ε and $\delta^{15}N$ are negligible compared to 1000, this equation can be rearranged to yield the final equation describing the evolution of the nitrogen isotopic enrichment in compartment i:

$$\frac{\mathbf{d\delta^{15}N_i}(t)}{\mathbf{dt}} = \sum_j \left[\frac{f_{i,j}(t)}{N_i} \times \left(\boldsymbol{\varepsilon}_{i,j} + \boldsymbol{\delta^{15}N_j}(t) - \boldsymbol{\delta^{15}N_i}(t)\right)\right] - \sum_j \left[\frac{f_{j,i}(t)}{N_i} \times \boldsymbol{\varepsilon}_{j,i}\right] \qquad \text{(E6)}$$

It should be noted that $\varepsilon_{j,i}$ is approximately equal to the difference between the isotopic enrichment of flux $f_{j,i}$ and that of its precursor compartment i, according to the following equation:

$\boldsymbol{\varepsilon_{j,i}} = \delta^{15}Nf_{j,i}(t) - \delta^{15}N_i(t)$, where $\delta^{15}Nf_{i,j}$ is the isotopic enrichment of flux $f_{i,j}$.
